# Management Protocol for Ballistic and Other High-Energy Avulsive Facial Injuries—An Update for the 21st Century

**DOI:** 10.3390/cmtr19010014

**Published:** 2026-03-03

**Authors:** Thomas Pepper, Michele H. Kim, Dane McMillan, Sarah Cantrell, Angel Scialdone, Angelina Nasthas, Ralph Erdmann, Paul N. Manson, David B. Powers

**Affiliations:** 1 Defence Medical Services, Guy’s & St. Thomas’ Hospitals and King’s College Hospital Major Trauma Centre, London SE5 9RS, UK; 2 Medical School, Duke University, Durham, NC 27710, USA; 3 Medical Center, Duke University, Durham, NC 27710, USA; 4 Library and Archives, Medical Center, Duke University, Durham, NC 27710, USA; 5 Department of Biomedical Engineering, Duke University, Durham, NC 27710, USA; 6 Johns Hopkins Medical Institute, Baltimore, MD 21205, USA

**Keywords:** ballistics, facial trauma, reconstruction, treatment protocol, avulsive facial wounds, high-energy wounds

## Abstract

High-energy ballistic and avulsive injuries to the face represent some of the most complex challenges in modern reconstructive surgery. Since Robertson and Manson’s 1999 management protocol, extensive military experience and technological advancements have transformed the treatment principles while preserving the core tenets of staged care. This updated review synthesizes evidence from 36 studies published since 2000, encompassing over two decades of global experience in both military and civilian trauma. Advances in damage-control resuscitation, wound decontamination, and early skeletal stabilization have improved survival and functional outcomes. Modern imaging—particularly intraoperative CT and navigation—enables the precise verification of the reduction and removal of retained fragments, while virtual surgical planning and patient-specific implants allow the accurate restoration of facial buttresses. Early vascularized tissue transfer has reduced contracture and infection rates. Adjuncts such as hyperbaric oxygen therapy, permissive hypotension, and advanced hemostatic agents further optimize recovery. The updated four-phase protocol—resuscitation, reconstitution, reconstruction, and rehabilitation—emphasizes early definitive repair, multidisciplinary collaboration, and the integration of digital planning. These refinements extend Robertson and Manson’s foundational principles into the era of precision surgery, achieving superior aesthetic and functional outcomes for patients with devastating facial injuries.

## 1. Introduction

In 1999, Robertson and Manson published the seminal paper “*High energy ballistic and avulsive injuries–a management protocol for the next millennium”* [1]. More than a quarter of a century has elapsed, with near-constant large-scale military conflicts in Afghanistan, Iraq, Ukraine, and Gaza. Now is the time to revisit the protocol, highlighting the advances from experiences gained in the interim. High-volume exposure to these injuries is historically isolated with regional and national variances with regard to civilian trauma centers in countries where firearms are readily available, locations with civil unrest, or military medical providers. Often, practitioners may go through an entire career without managing these injuries, while other surgeons may see these wounds on a weekly, or near-constant basis.

The head, face, and neck are, unfortunately, common battlefield injuries, comprising over 20% of injuries in US and UK military personnel serving in Iraq and Afghanistan and approximately 25% in Ukraine [2,3,4,5]. Improvements in body armor have allowed personnel to recover from previously lethal torso trauma, but the face remains relatively unprotected [6,7].

Robertson and Manson’s article describes these overlapping phases for the management of high-energy ballistic and avulsive facial injuries [1]:

1. Phase I—Resuscitation: 3D CT scanning, and damage control including the excision of the zone of tissue necrosis but with the judicious debridement of the zone of injury, followed by serial wound re-exploration and debridement every 24–36 h;
2. Phase II—Wound stabilization and reconstruction of the functional units of the upper and lower facial halves with unification at the Le Fort I level;
3. Phase III—Reconstruction of missing mucosal lining, bone, and cutaneous soft tissue, ideally within 5–7 days of injury (to avoid scar contracture). The upper face may be reconstructed with non-vascularized bone, whereas the lower face may require free-tissue transfer. Once the reconstruction is stable, a secondary revision using local tissue may be performed.

The original paper encouraged surgeons to move away from cautious traditional methods of management, with delayed reconstruction, and, instead, favor treatment with earlier internal fixation, while urging close attention to wound bed preparation so that early reconstruction has a higher chance of success. High-energy ballistic injuries to the craniomaxillofacial region create complex, contaminated composite defects with an evolving zone of injury and high risk for infection, soft-tissue loss, and functional compromise. Contemporary management generally applies damage-control principles, early infection control and wound optimization, and definitive reconstruction using modern fixation, microvascular techniques, and virtual surgical planning when appropriate. However, the literature remains heterogeneous and largely limited to case series, with inconsistent definitions and outcome reporting, leaving persistent uncertainty regarding the optimal timing and sequencing of reconstruction, objective debridement endpoints, and comparative outcomes across reconstructive strategies. This study addresses these gaps systematically, reviewing current approaches using standardized categories for injury characteristics, reconstruction timing, and complication definitions, and by proposing a pragmatic, reproducible decision framework to guide clinical reporting and management.

## 2. Pathophysiology of the High-Energy Ballistic Wound

An understanding of the pathophysiology of ballistic injury, and the fact this entity is not limited to injuries caused by firearms, is necessary to appreciate the design of the study. All weapons causing the flight of one or more projectiles in a ballistic fashion (i.e., moving solely under the influence of gravity after launch) may cause this injury type. In a military setting, this includes shrapnel from artillery strikes, improvised explosive devices, drones, rockets, and bombs. Such injury may be further complicated by a superimposed blast injury. In a civilian setting, non-firearm ballistic injuries have been reported from airbags, slingshots, propane tank explosions, nail-guns, and crossbows [8,9,10,11]. Gunshot wounds are themselves a heterogeneous group of ballistic injuries, and vary widely depending on the weapon, type of ammunition, compositional makeup of the projectile, energy transfer, range, missile trajectory, and personal protective equipment worn by the victim [12].

**Figure 1:**
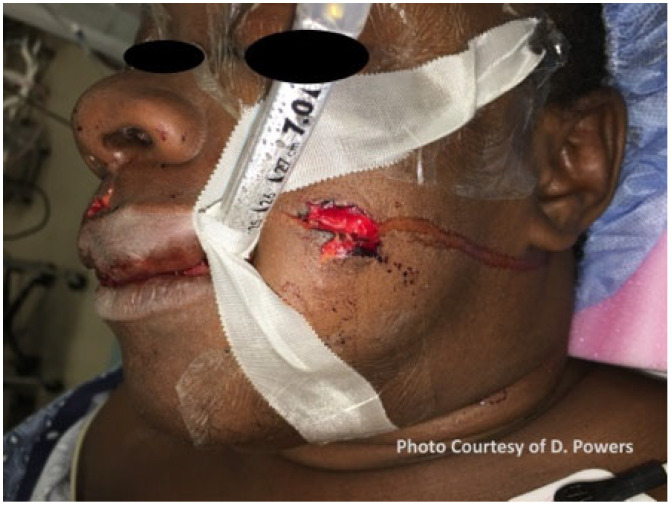
Low-energy gunshot wound. Note the lack of significant soft-tissue disruption.

**Figure 2:**
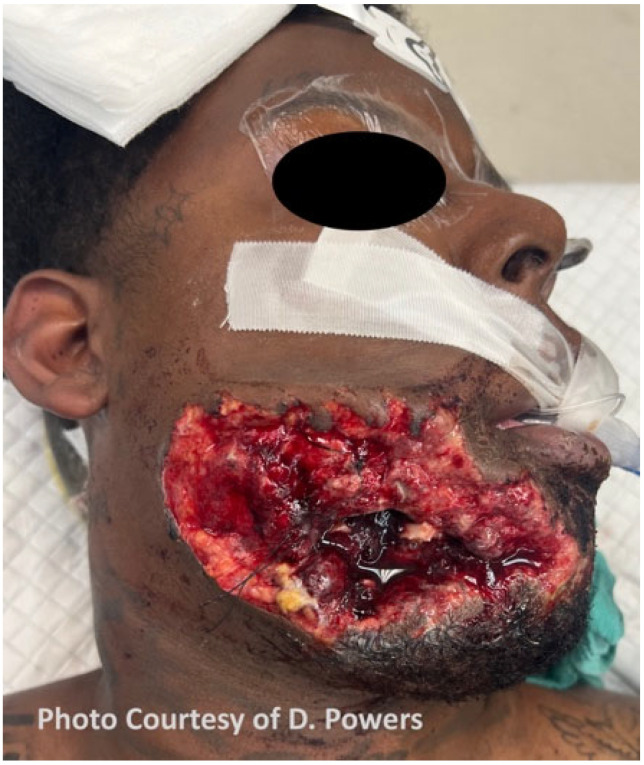
High-energy gunshot wound with associated avulsive loss of soft tissue and bone.

The definition of a “high-velocity” ballistic wound is nebulous and was historically characterized by the muzzle velocity of the weapon used. In American literature, a high-velocity ballistic weapon is classified as having a velocity of 2000–3000 feet per second, while, in British literature, it is classified as having a velocity of 1125 feet per second, which is the speed of sound in air. The exact cut-off is perhaps academic, as the offending weapon type is frequently unknown and energy transfer is subject to the factors previously mentioned. Due to these and other variances and inconsistencies with the definition, the word “velocity” should no longer be used to describe ballistic wounds, and the terms “high-energy” and “low-energy” are more accurate in describing wounding patterns. In broad terms, an injury resulting from a handgun is typically accepted as being low-energy, with the exception of weapons that utilize higher-volume propellant (Magnum) loads. In contrast, an injury resulting from a military or hunting rifle would be considered high-energy. A hallmark of high-energy ballistic injury is significant tissue avulsion or displacement, requiring more specialized treatment. On the other hand, low-energy ballistic trauma can frequently be managed similarly to blunt injury. Information about the characteristics of contemporary ballistic missiles, including shotgun projectiles, is relevant to this topic but has been covered elsewhere and is outside the scope of this paper [13,14] (Figure 1 and Figure 2).

**Figure 3:**
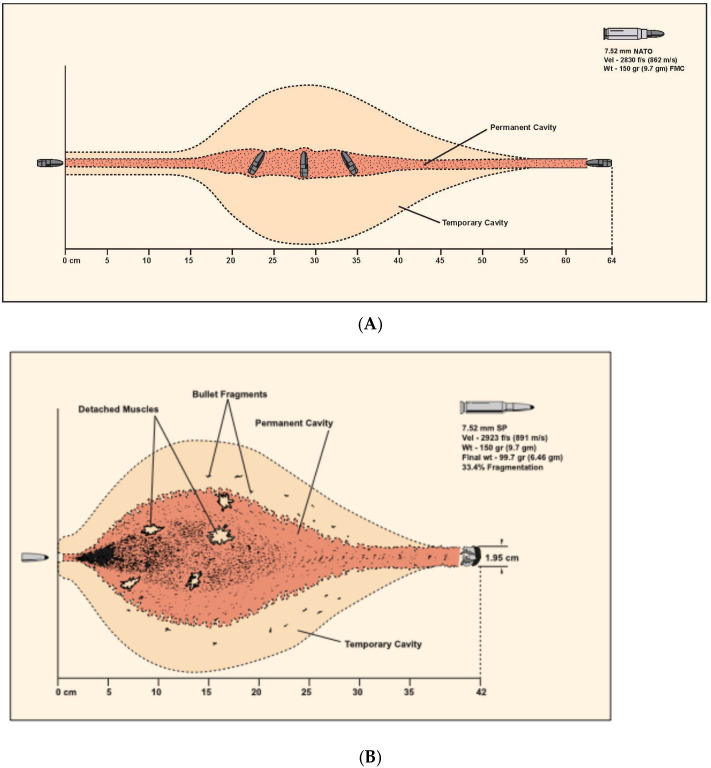
(**A**) Ballistic representation of NATO 7.52 mm round fired from a rifle. Observe the relatively consistent permanent cavity and laterally radiating temporary cavity, which begins to develop at approximately 20 cm into the tissue as the projectile begins to tumble. This chart represents the projectile not striking any hard structures causing deformation or alteration in trajectory. The anatomic characteristics of the head and neck do not have more than 20 cm of soft tissue present before encountering the bony skeleton, which would have a clinical significance with regard to the temporary cavity should the projectile be of a trajectory to encounter only soft tissue and miss the underlying facial bones. (**B**) Ballistic representation of a 7.52 mm soft-point (SP) round striking muscle and bone. Note, as the projectile strikes the underlying structures, there is a tremendous increase in the permanent cavity, as well as the temporary cavity, as the projectile deforms and fragments because of the soft-tip construction. This deformation in the structural characteristic of the projectile, and associated increase in the permanent and temporary cavities, greatly enhances the wounding potential of this round. *Modified From: Emergency War Surgery. 5th edition, Washington DC, USA Government Printing Office, 2013*.

Ballistic wounds have been conceptualized as containing a zone of tissue loss, the permanent cavity, and a zone of tissue injury, the temporary cavity (Figure 3). The temporary cavity is further subdivided into an inner zone of extravasation/contusion and an outer zone of concussion [14]. Tissue crush is responsible for the zone of loss, while tissue stretch causes the zone of injury. The degree of damage in the zone of injury depends upon the types of tissues in that area. Elastic tissues, such as muscle, are relatively resistant to damage by stretch, whereas inelastic tissues, such as bone and, to a lesser extent, blood vessels, are more prone to damage. In the days following the wound, the zone of injury demarcates into necrotic tissue that becomes part of the permanent cavity, and healing tissue that eventually recovers. There is controversy over the size of the zone of injury, as the temporary cavity in ballistic gelatin is larger than has been observed in vivo [15,16,17]. This has led to a variety of myths, not least that the size of the temporary cavity may be thirty times the size of the projectile, and that cell death can occur as much as 20 cm from the edge of the bullet hole, requiring the debridement of this entire area. While these extreme contentions have been refuted, in the craniomaxillofacial region, the situation differs further [18]. There is a relatively thin soft-tissue envelope overlying the facial skeleton, which is generally less than the distance, approximately 25 cm, required for a full metal-jacketed projectile to cause temporary cavitation [13]. Thus, for craniomaxillofacial injuries, the extent of injury is primarily related to the characteristics of the tissues involved, the velocity of the projectile at impact, and the degree of the yaw, deformation, and fragmentation of the projectile as it travels through the tissues, and debridement can be much more judicious than some authors have historically advised [15,19,20].

For clarity, high-energy injuries are defined herein as ballistic trauma with significant energy transfer resulting in comminution, avulsion, cavitation injury, or volumetric tissue loss, regardless of projectile velocity. Since the original publication of the Robertson and Manson article, numerous publications and advancements have occurred in the surgical treatment of high-energy ballistic injuries to the craniomaxillofacial region. The purpose of this study is to review the literature since the original presentation of this article to provide an updated management protocol for treating high-energy ballistic and avulsive injuries to the face, building upon the primary work of Robertson and Manson. The specific aim is to provide an updated management protocol, contemporary to today’s technological and surgical advances, thereby improving the treatment of patients with these injuries.

## 3. Methods

### 3.1. Eligibility Criteria

Inclusion criteria included the following: human studies reported in randomized controlled trials, cohort studies, case–control studies, and case series with five or more patients, published in the English language, describing the management of gunshot wounds/ballistic trauma, providing outcomes data related to postoperative complications and/or aesthetic outcomes, published in the year 2000 or after.

Exclusion criteria include the following: non-English language papers, animal studies, in vitro studies, publications before the year 2000, studies not separately reporting outcomes of ballistic trauma from other forms of trauma, reports with fewer than five patients, literature reviews, letters to the editor, conference abstracts, review articles, and opinions.

Although the focus of this review is on high-energy ballistic and avulsive maxillofacial trauma, the authors intentionally did not restrict the literature search to only those studies explicitly labeled “high-energy.” This decision was made because the vast majority of published ballistic facial trauma studies do not classify energy transfer in a standardized manner, and many otherwise highly relevant investigations fail to distinguish between high- vs low-energy mechanisms in their title, abstract, or methods. Restricting the search solely to high-energy terms would therefore have excluded important cohorts that clearly involved avulsive and high-energy ballistic injury patterns upon full-text review. To ensure completeness, the authors applied high-energy inclusion criteria during the data extraction and study selection phase, resulting in a final dataset that reflects the intended population, even if initial abstracts did not explicitly use this terminology.

### 3.2. Information Sources and Search Strategy

The MEDLINE (Ovid), Embase (Elsevier), and Cochrane Library (Wiley) databases were searched from 2000 to present using a mix of database-specific controlled vocabulary terms and keywords searched in the title or abstract for literature on gunshot wounds (including war, conflict, and regional injury terms) and craniomaxillofacial terms. An experienced medical librarian (S.C.) devised and conducted the searches, with input on keywords from the senior author (D.P.). Animal studies, editorials, letters, commentary, conference abstracts, and articles not in English were excluded. The original searches were conducted on 4 March 2024. The searches were independently peer-reviewed by a librarian using a modified PRESS Checklist [21].

### 3.3. Selection Process

The search included studies published between 2000 and May 2025. All records were imported into Covidence (Version 2668; Covidence, Melbourne, VIC, Australia). Primary title and abstract screening were undertaken using the eligibility criteria below. Four reviewers (M.K., R.E., A.S., and A.N.) independently screened titles and abstracts in the Covidence systematic review screening software. A designated third-person arbitrator resolved all conflicts. Titles and abstracts that met the eligibility criteria were retrieved and assessed fully. Full-text studies were, once again, independently screened by two reviewers, with any conflicts resolved by the designated third reviewer. Full-texts not meeting the eligibility criteria at this stage were recorded and excluded.

### 3.4. Data Collection Process and Items

**Figure 4:**
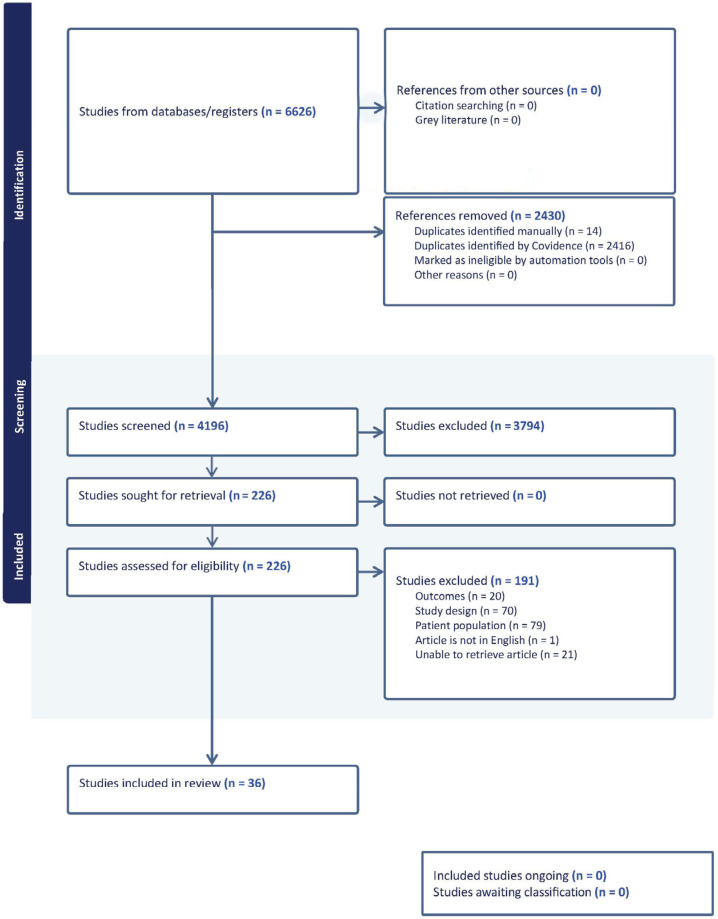
PRISMA Diagram of Research Study Protocol.

Study identification, screening, and selection followed the Preferred Reporting Items for Systematic Reviews and Meta-Analyses (PRISMA) guidelines. All citations retrieved from the database search were imported into Covidence for deduplication, independent screening, data extraction, and review management. Discrepancies at any stage were resolved through consensus with a third reviewer. The PRISMA flow diagram (Figure 4) summarizes the number of records identified, screened, excluded, and included, with reasons for exclusion documented at the full-text review stage. A standardized data extraction template was developed a priori. The following data items were collected from each eligible study:

- **Study characteristics:** Authors, publication year, country/region, study design, and setting (civilian vs battlefield);
- **Population details:** Sample size, age when available, and mechanism and **energy profile** of ballistic injury;
- **Injury characteristics:** Anatomic location(s), presence of avulsive defects, and retained projectiles;
- **Management variables:** Timing of intervention (categorized as immediate <48 h, early ≤30 days, delayed >30 days, or not reported), fixation/reconstruction techniques, and use of image guidance or patient-specific hardware;
- **Outcomes:** Bony union, soft-tissue healing, complications (e.g., infection, and flap failure), and aesthetic and functional results when reported.

These data elements were selected to directly address the review objectives related to timing and methods of reconstruction in high-energy ballistic and avulsive craniomaxillofacial trauma. When timing variables were not explicitly reported, studies were included for qualitative outcome assessment but excluded from quantitative timing analysis.

## 4. Results

A total of 6626 unique citations were identified after the removal of duplicates (Figure 4). Following the title and abstract screening and full-text review in Covidence, 36 studies met the eligibility criteria and underwent data extraction (Supplementary Table S1). The included studies comprised 3 cross-sectional studies, 10 retrospective cohort studies, 1 retrospective case–control study, 11 case series, 2 randomized controlled trials (RCTs), and 9 retrospective chart reviews. The mean age of the patients, when reported, ranged from 16.5 to 53 years. The firearms included handguns, pistols, assault rifles, shotguns, and improvised or homemade weapons. The differences between this study and the findings of the original Manson and Robertson article are summarized in Table 1.

The timing of intervention was codified as per Vaca et al., with immediate debridement and definitive reconstruction being performed within 48 h of injury, early definitive reconstruction occurring within 30 days of injury, and delayed definitive reconstruction performed past 30 days of injury [22]. Of the studies originating in the Middle East, two of the eight advocated for immediate intervention, while two of the eight studies reported early reconstruction [23,24,25,26]. One study advocated for delayed reconstruction; Guerrier et al. reported a range of 45 days to 10 years as they reconstructed mandibular defects using iliac crest bone grafts [27]. Three of the eight studies did not report their timing of intervention [18,19,20,21,22,23,24,25,26,27,28,29,30].

Twelve studies were set in the United States and had various intervention timings. Three studies involved immediate surgical intervention, and four studies were performed within one week of injury (early surgical intervention) [31,32,33,34,35,36,37]. All other studies did not report the timing of surgical intervention [38,39,40,41]. One study advocated for a staged treatment protocol, initially debriding and stabilizing the fracture, and then following up with secondary, definitive procedures, such as fracture repair with bony reconstruction [42].

Out of the five studies in Turkey, one advocated for immediate debridement and reconstruction with a plate, then staged reconstruction [24]. Another study utilized free flaps performed within two months for all patients [43]. Lastly, Vayvada et al. compared immediate to delayed reconstructive free flap treatment and reported lower infection, lower scarring rate, and fewer deformities from tissue contraction in the immediate reconstruction cohort [44]. Two studies did not report any intervention timing for the repair of military maxillofacial injuries [45,46]. 

Rana et al. and Channar et al. both advocated for the early definitive repair of mandibular fractures, with open reduction and internal fixation (ORIF), reporting better outcomes due to improved bone stabilization, good functional and aesthetic results, and a low complication rate [47,48]. Siddiqui et al., despite not reporting the timing of their intervention, advocated for ORIF as well, instead of maxillomandibular fixation (MMF), due to the improved healing [49]. However, Muddassar et al. reported higher infection rates with ORIF but a lower non-union rate; this study did not report the timing of their intervention [50]. Two other studies set in Pakistan did not report the timing of the intervention [51,52].

Jose et al. and Jeyaraj et al. both advocated for immediate, definitive reconstruction for isolated maxillofacial injuries [53,54]. When comparing patients treated with early, aggressive, surgical intervention through ORIF to patients managed conservatively with delayed repair, early repair showed better healing and less complex surgical revisions.

Gröbe et al. utilized image guidance to remove surgical projectiles and utilized early definitive reconstruction. While the timing of intervention was not recorded, the immediate reconstruction of defects was reported as being as functionally and aesthetically effective as routine reconstruction procedures. Image guidance reportedly lowered complication rates, such as significant bleeding, soft-tissue infection, and nerve damage [55]. Xing et al. also studied image guidance for endoscopic projectile removal and reported improved wound healing with similar findings to Gröbe et al [56]. Wei et al. reported improved outcomes when patients received debridement and the removal of foreign bodies under stereotactic or neuronavigational guidance within 8 h of injury. Immediate removal was found to protect tissue, stop bleeding, and improve aesthetics [57].

Fifteen studies reported no definitive timing of the intervention. Out of the studies that reported the timing of reconstruction, 10 of the 20 (50%) advocated for immediate intervention, 8 of the 20 (40%) advocated for early intervention, and 2 (10%) reported data from delayed intervention. Two of the three studies advocating delayed reconstruction were from the Middle East (Turkey and Iraq), whereas seven of the eighteen studies advocating immediate or early definitive reconstruction were from the United States. Of the studies that reported the setting of the injury, self-inflicted injuries were responsible for 5.0% (109 of 2169) of wounds, military injuries for 40.2%, and civilian non-self-inflicted injuries for 54.8%.

## 5. Discussion

This protocol update is based on a systematic narrative review of predominantly Level III–IV evidence in combination with the senior authors’ and co-authors’ clinical experience in both civilian and battlefield settings. Where possible, recommendations are supported by published data; in areas where the literature is sparse or heterogeneous, they represent the expert consensus derived from contemporary practice in high-volume craniomaxillofacial trauma centers and military medical systems. Recommendations were categorized as evidence-supported (Level III); case-series-supported (Level IV), with the appropriate citation of the published work; or experience-informed, where higher-level data were unavailable, reflecting the ethical and logistical constraints of ballistic trauma research.

### 5.1. Phase I—Resuscitation and Damage Control Surgery

#### 5.1.1. Bleeding

At the turn of the millennium, it would have been unthinkable in a discussion of resuscitation to consider bleeding in advance of airway management, but 21st-century battlefield experience determined catastrophic hemorrhage to be the most common cause of potentially survivable combat death, and, thus, ABC evolved to **<C>ABC** (Catastrophic Hemorrhage—Airway, Breathing, Circulation) or **MARCH** (Massive Hemorrhage, Airway, Respiration, Circulation, Hypothermia, and Head Injury) in battlefield resuscitation algorithms [58,59,60,61,62,63]. This change is gradually being reflected in civilian major trauma protocols, focusing on exigent volume replacement in concert with airway securement [64].

**Figure 5:**
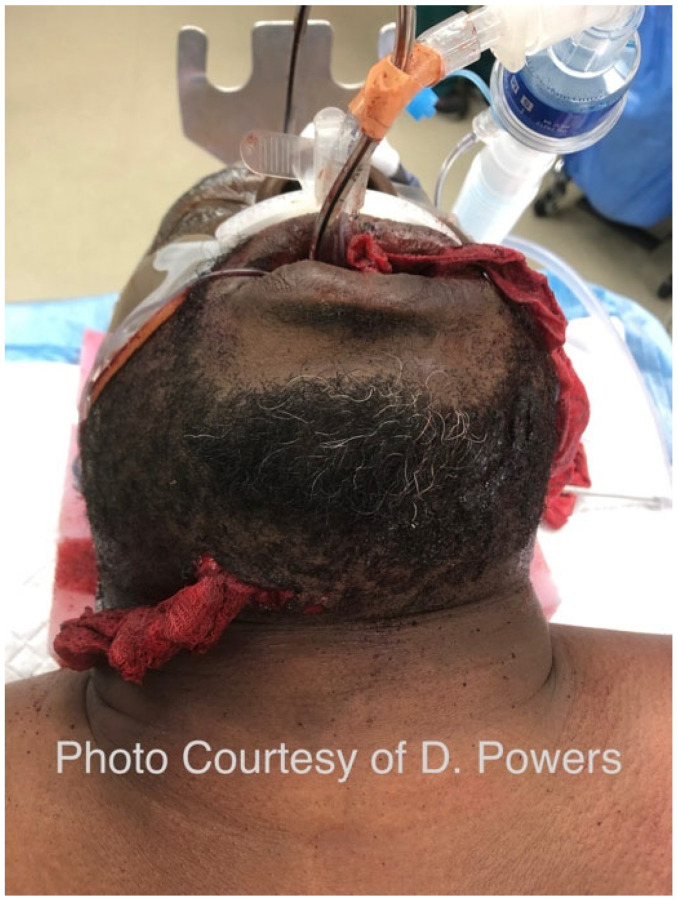
Hemostatic gauze used to control bleeding from transected lingual artery after a self-inflicted gunshot wound.

With the recognition of the role of catastrophic hemorrhage in avoidable deaths came a resurgence in tourniquet use in the extremities, but, for head and neck hemorrhage, these are clearly unsuitable [65] A renewed imperative in research for agents to arrest junctional hemorrhage has yielded advances in topical hemostatic agents, with chitosan-based (*HemCon*^®^, Tricor Biomedical, Portland, OR, USA, and *Celox Rapid*^®^, Med Trade Products Limited, Crewe, UK) and kaolin-based (*QuikClot Combat Gauze*^®^, Z-Medica, Wallingford, CT, USA) products being adopted for use by a range of military combat and medical forces, with a proven record of establishing hemostasis on critically wounded personnel in combat operations [66,67]. This record of success has resulted in the expansion of this material to civilian pre-hospital use, and hemostatic products are reportedly now carried by 47% of Emergency Medical Services [68,69,70] (Figure 5).

Trauma-induced coagulopathy (TIC) is present in 1 in 4 major trauma patients at admission and is associated with a three-to-fivefold increase in mortality [71,72,73]. Historically, INR > 1.5 and platelets < 100,000 μL have been used to define the presence of coagulopathy; however, these tests have a prolonged laboratory turnaround time; therefore, newer tests have been developed. Thromboelastography (TEG) and rotational thromboelastometry (ROTEM) have shown promise and have been used to reduce hemorrhage and guide massive transfusion in cardiovascular surgery and liver transplantation. More recently, these tests have also been applied to trauma [74]. Tranexamic acid is a synthetic antifibrinolytic drug that inhibits the degrading action of plasmin on fibrin clots. Its effectiveness at reducing mortality in trauma patients has been proven in both military and civilian settings [75,76]. It has been shown to reduce blood loss irrespective of the type of surgery, and, for this reason, has become widely incorporated into both trauma and elective surgery protocols [77,78,79].

The last two decades have seen a paradigm shift in the type of fluid used for trauma resuscitation. For the initial resuscitation fluid, there has been a widespread shift from colloid to crystalloid, but the benefits of limiting even this administration have been recognized [80]. The goal currently is permissive hypotension, in an effort to minimize exsanguination and the early transfusion of blood products. A similar transition has been underway for the transfusion of blood products, from component therapy in unbalanced ratios, to a 1:1:1 transfusion ratio, and, more recently, to the use of warm, fresh whole blood [81]. Once again, this latter advance came as a product of war, with an improvement in 30-day survival noted in patients with combat-related traumatic injuries who were treated with whole blood [82]. This survival benefit has since been successfully applied to civilian major trauma in a growing number of centers, with the use of whole blood as an adjunct to component therapy improving survival within 5 h of ED presentation and lowering the risk of mortality by 37% at 24 h [83]. 

There is a 10–50% incidence of major vascular injury in facial gunshot wounds [84]. Hence, the above measures may be a means of increasing patient survival, enabling patients to reach the operating room for the vascular control component of damage control surgery. Damage control resuscitation, however, continues throughout, and consultation with the anesthetic and hematology teams will inform ongoing blood product use. At the same time, the surgeon identifies and controls the source, or sources, of bleeding.

Consensus Recommendation: The contemporary management of ballistic craniomaxillofacial trauma should prioritize catastrophic hemorrhage control as the initial resuscitative step, reflecting the evolution from traditional ABC algorithms to <C>ABC/MARCH principles derived from battlefield experience. The early use of topical hemostatic agents, balanced or whole-blood-based damage control resuscitation, viscoelastic-guided correction of trauma-induced coagulopathy, and the timely administration of tranexamic acid are recommended to reduce preventable mortality and enable definitive surgical hemorrhage control. These measures should be integrated into civilian trauma protocols through coordinated multidisciplinary resuscitation extending from the prehospital setting to the operating room.

#### 5.1.2. Airway

Airway obstruction was the third leading cause of potentially survivable combat death, after hemorrhage and tension pneumothorax, in the US Special Operations Forces [58]. High-energy penetrating head and neck injuries have a close association with airway embarrassment. In Brennan et al.’s 2011 review of 196 trauma patients with airway compromise during Operation Iraqi Freedom, 186 airway interventions were surgical airways, 68% of which were considered potentially lifesaving [85]. The most common (75%) etiology of airway compromise was penetrating face and neck trauma. While, occasionally, avulsive injury to the neck can expose the larynx, resulting in a traumatic laryngostomy, in which case the optimal initial treatment is to sit the patient forward to allow secretions to drain away from the exposed airway, more commonly, airway stabilization in the form of endotracheal intubation or tracheostomy is required.

Keller et al. reviewed 239 combat maxillofacial injuries sustained either through a blast or gunshot between 2004 and 2010 in Iraq and Afghanistan, and found that 19.3% underwent tracheostomy, the majority within 48 h of injury [86]. Injury by a gunshot mechanism was associated with an increased need for tracheostomy (*p* = 0.03). After adjusting for the injury severity and mechanism of injury, military personnel with a facial fracture had higher odds of undergoing tracheostomy (OR 4.1, 95% CI 1.3–13.2) [86].

Consensus Recommendation: Given the high incidence of airway compromise in ballistic craniomaxillofacial trauma and its association with preventable mortality, early and proactive airway management should be a priority following hemorrhage control. High-energy penetrating injuries to the face and neck warrant a low threshold for definitive airway stabilization—often via early tracheostomy—particularly in gunshot-related facial fractures, where delayed airway intervention is associated with increased risk. Multidisciplinary trauma teams should anticipate airway deterioration and intervene early to ensure safe resuscitation and operative management.

#### 5.1.3. Eyes

**Figure 6:**
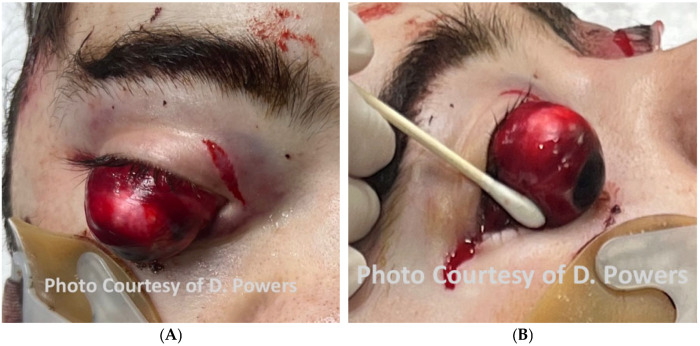
(**A**) and (**B**) Globe subluxation associated with bitemporal injury from self-inflicted gunshot wound.

**Figure 7:**
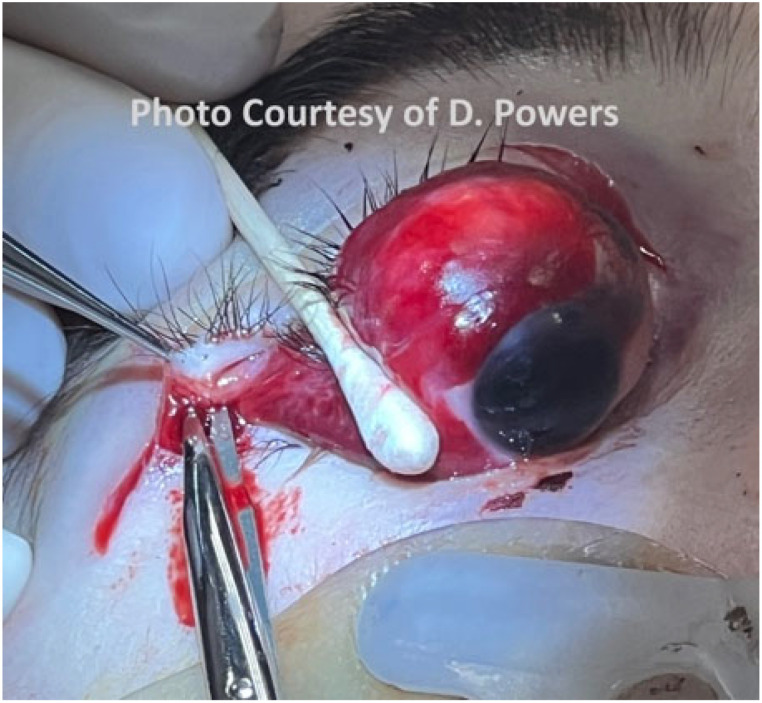
Canthotomy and reduction of the subluxated globe.

**Figure 8:**
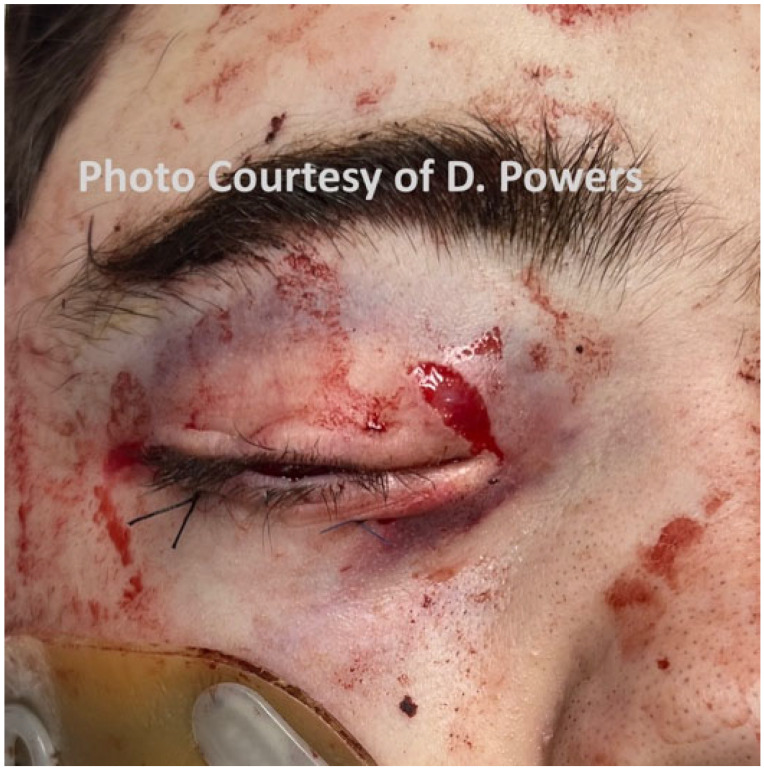
Globe after reduction and stabilization of reinsertion with tarsorrhaphy sutures.

The comprehensive management of ocular injuries is beyond the scope of this article, but injuries to the orbits should be assessed in Phase I, and sight-saving interventions made as necessary. The evaluation will involve the clinical assessment of retrobulbar hemorrhage and its timely treatment via lateral canthotomy and cantholysis [87]. Globe luxation injuries should be managed acutely by gently returning the globe to the orbit via the techniques described in the literature, followed by the placement of tarsorrhaphy sutures to maintain its position [88,89] (Figure 6A,B and Figure 7 and Figure 8). Traumatic luxation injuries carry a poor prognosis for vision, but functional recovery has been reported up to a week post-luxation [88]. Open globe injuries merit specialist ophthalmological management. In a permissive environment, primary surgery should ideally take place within 24 h, but, for a polytrauma patient in an austere environment, achieving ophthalmic surgery within this timeframe is likely to be unrealistic [89,90,91,92].

Consensus Recommendation: In ballistic craniomaxillofacial trauma, orbital injuries should be identified and addressed early during Phase I evaluation, with priority given to sight-saving interventions such as the prompt recognition and decompression of retrobulbar hemorrhage and the appropriate acute management of globe luxation. While definitive ophthalmologic repair—particularly for open globe injuries—should ideally occur within 24 h in permissive settings, management strategies must remain adaptable in remote or polytrauma environments, emphasizing timely stabilization, the protection of ocular structures, and early specialist consultation when feasible. In an austere environment, medical personnel should have additional training in the management of ocular injuries, specifically the capacity to perform orbital decompression.

#### 5.1.4. Infection

**Figure 9:**
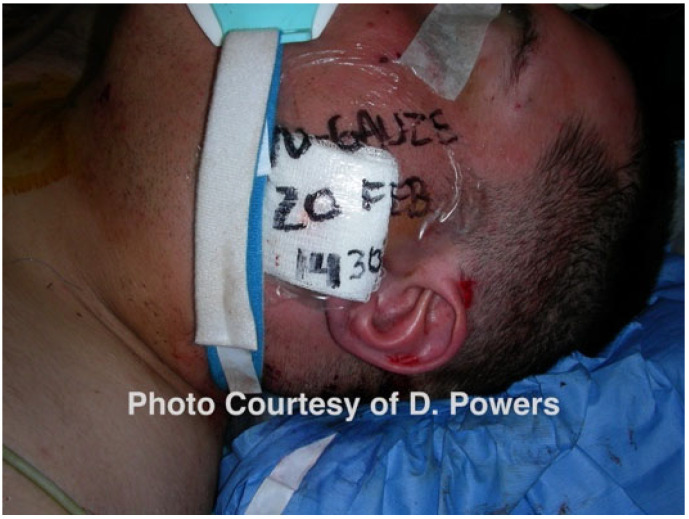
Marking of wound dressing to alleviate confusion of treatment personnel for the date and time of treatment.

The urban myth that a bullet is a sterile projectile has been roundly disproven [93]. All battlefield and ballistic injuries should be considered contaminated, and, if encountered in a setting of warfare, when combined with the patient’s compromised nutritional state, likely sleep deprivation, and high-stress environment, predispose patients to a high rate of infection [94]. Oral and maxillofacial surgeons pioneered the pulsed lavage of wounds during the Vietnam War, and this has since become a conventional strategy in the extremities and torso; however, its use by civilian head and neck surgeons is rare [95]. While there has been some caution regarding the potential of high-pressure lavage to seed bacteria into deeper tissues, its overall effectiveness at significantly reducing surgical site infections is undisputed [96,97,98]. Successful use at low to medium settings during military deployments indicates the acceptance of this treatment modality in the combat care protocols of military healthcare organizations [99,100]. Following their experience in Operation Iraqi Freedom, Will et al. recommended a regimen of high-volume lavage followed by the temporary packing of deep wounds with a single length of iodoform gauze. The surgeon must bear in mind that, frequently, these packs may not be removed until the patient arrives at the next level of care, and marking the date/time of placement is recommended [94]. Marking the dressing change in a civilian setting also eliminates confusion with the provider and nursing staff turnover and clinical-shift differentials (Figure 9).

The infection of wounds with multi-resistant *staphylococci*, *enterococci*, and *clostridia* has been a frequent feature of recent wars. *Acinetobacter baumanii* infection characterized injuries from Iraq and Afghanistan and a similar picture has emerged from Ukraine [101,102]. At the point of arrival at the next level of care, the removal of packs and a “second look” procedure for further irrigation and judicious debridement are mandatory, rather than performing immediate primary closure [94,103]. This process should be repeated every 24–36 h until the wound bed is considered clean.

Consensus Recommendation: All ballistic craniomaxillofacial wounds should be considered contaminated and managed with an infection-mitigation strategy that emphasizes early, aggressive irrigation and staged debridement rather than primary closure. High-volume, low- to medium-pressure pulsed lavage followed by temporary wound packing is recommended to reduce the bacterial burden, with the clear labeling of dressings to ensure the continuity of care across treatment settings. Given the high incidence of multidrug-resistant organisms in ballistic injuries, mandatory re-exploration with repeat irrigation and judicious debridement at subsequent levels of care is essential until a clean, viable wound bed is achieved. Based on the customary bacterial contaminants isolated in ballistic injuries in a civilian or military environment, the initial prophylaxis recommendations would consist of cefazolin, ampicillin/sulbactam if the respiratory sinuses are involved, or clindamycin. In circumstances where surgical therapy may be delayed, consideration should be given to ertapenem or moxifloxacin.

#### 5.1.5. Transition to Phase II

After Phase I treatment, the patient should have a secure airway in place, with no ongoing hemorrhage, and with ballistic wounds that have undergone judicious decontamination/debridement, irrigation, and packing. The patient should be physiologically stable or improving following resuscitation efforts, with the correction of biochemical abnormalities, and antimicrobial coverage. Ballistic injuries sustained in an austere environment, such as a theater of war or remote hunting location, may require additional considerations of “packaging” the patient for transport by off-road, sea, or air. For panfacial or polytrauma patients, this often means a pre-emptive tracheostomy [94]. Once the patient is ready for Phase II treatment, if a tracheostomy is not already in place and is not deemed necessary in the foreseeable future, a submental intubation provides a low-morbidity alternative route for the endotracheal tube, allowing for full surgical access to the mandible and midface should the naso-endotracheal intubation be contraindicated.

### 5.2. Phase II—Wound Stabilization and Reconstitution

Phase II treatment involves wound stabilization and reconstitution. “Reconstitution,” rather than “reconstruction,” is the term used to imply the use of existing local tissues to rigidly stabilize the buttresses of the facial skeleton and maintain the soft-tissue envelope, rather than the imported tissues of the next “reconstructive” phase.

#### 5.2.1. Stabilization of the Wound Bed

**Figure 10:**
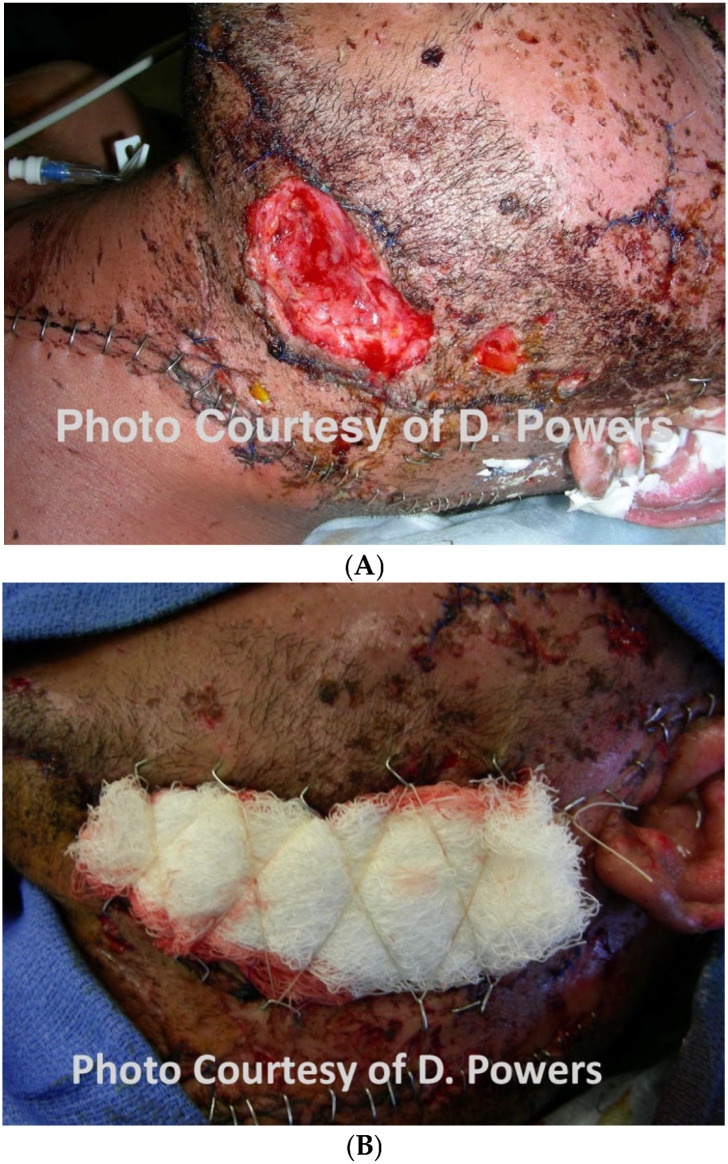
(**A**) Soft-tissue wound from improvised explosive device with defect that is not amenable to immediate reconstruction secondary to associated tissue injury to surrounding tissues. (**B**) Same wound after temporizing with a wound dressing.

The management of the wound bed that commenced in Phase I continues into Phase II, and irrigation and debridement should be performed every 24–36 h until the wound is clean on review. At this point, the best available closure that does not significantly distort the soft tissues is accomplished. Areas that cannot be closed are left packed in anticipation of Phase III (Figure 10A,B).

**Figure 11:**
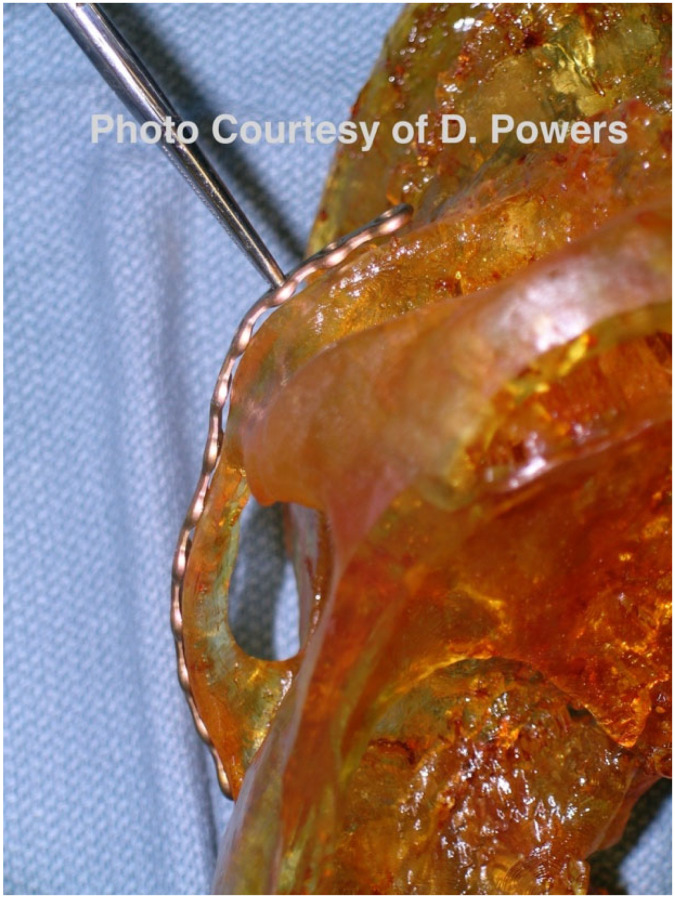
Pre-bending of plates to fabricated stereolithography model to assist with anatomic reconstruction of the injured, contralateral zygomatic complex.

Many patients will have already undergone cross-sectional imaging by this phase, but this is likely to be a low-resolution trauma scan evaluating the cervical spine and cranium. Contemporary computed tomography (CT) scanners can perform “fine cuts” with a thickness of 0.625 mm, allowing for reformatting into a high-resolution 3D digital model that aids in an enhanced understanding of the spatial relationships of craniomaxillofacial injuries and retained projectiles. Imaging may be combined with appropriate software to allow the use of intraoperative navigation, which facilitates the subsequent treatment stages. If such a scan is not already available, it is prudent to obtain one before embarking on further treatment. Three-dimensional printing has come of age in the 21st century, and several military authors have championed the benefits of stereolithographic models derived from digital imaging and communication in medicine (DICOM) data obtained during CT scans to both facilitate treatment planning and allow faster operating through the pre-adaptation of plates [4,99] (Figure 11).

Consensus Recommendation: The Phase II management of ballistic craniomaxillofacial injuries should focus on wound stabilization and skeletal reconstitution using available local tissues, with continued serial irrigation and debridement every 24–36 h until a clean wound bed is achieved. Definitive but conservative closure should be performed only when it preserves the soft-tissue envelope and skeletal buttresses, while unresolved defects are temporized in anticipation of later reconstruction. High-resolution CT imaging, with the optional use of three-dimensional modeling and navigation when available, is recommended at this stage to optimize spatial understanding, facilitate plate adaptation, and improve efficiency and accuracy in subsequent reconstructive phases.

#### 5.2.2. External Fixation

**Figure 12:**
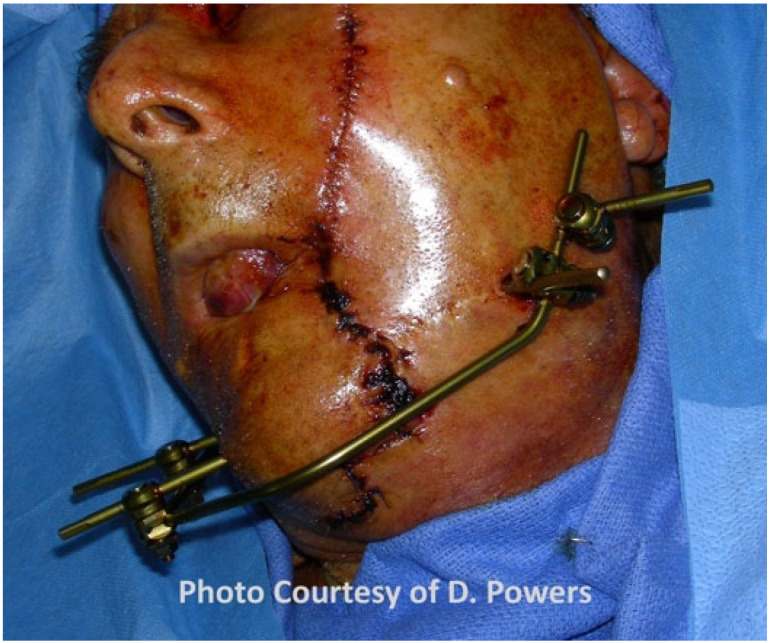
External fixation of a comminuted mandibular fracture from a gunshot wound.

While external fixation has fallen out of favor for low-energy ballistic injuries, military experience has proven it to be expeditious in managing high-energy and avulsive mandibular injuries as it allows the stabilization of the bone fragments and the maintenance of the soft-tissue envelope dimensions, thereby minimizing scar contracture [12] (Figure 12).

Consensus Recommendation: The use of external fixation can be a valuable resource for the management of ballistic injuries, specifically as a temporizing measure prior to definitive reconstruction. Care should be taken when utilizing this modality for avulsive wounds as irreversible scar contracture can occur in the region of hard tissue loss. The clinical adaptability of this treatment modality affords for non-conventional uses, such as external fixation being utilized to perform the immobilization of the maxillomandibular complex for the stabilization of corticocancellous graft placement when the patient is missing stable dentition by securing the zygoma to the mandible. Additionally, the cross-face soft-tissue projection of a comminuted zygomaticomaxillary complex has also been successfully utilized with external fixation.

#### 5.2.3. Internal Fixation

**Figure 13:**
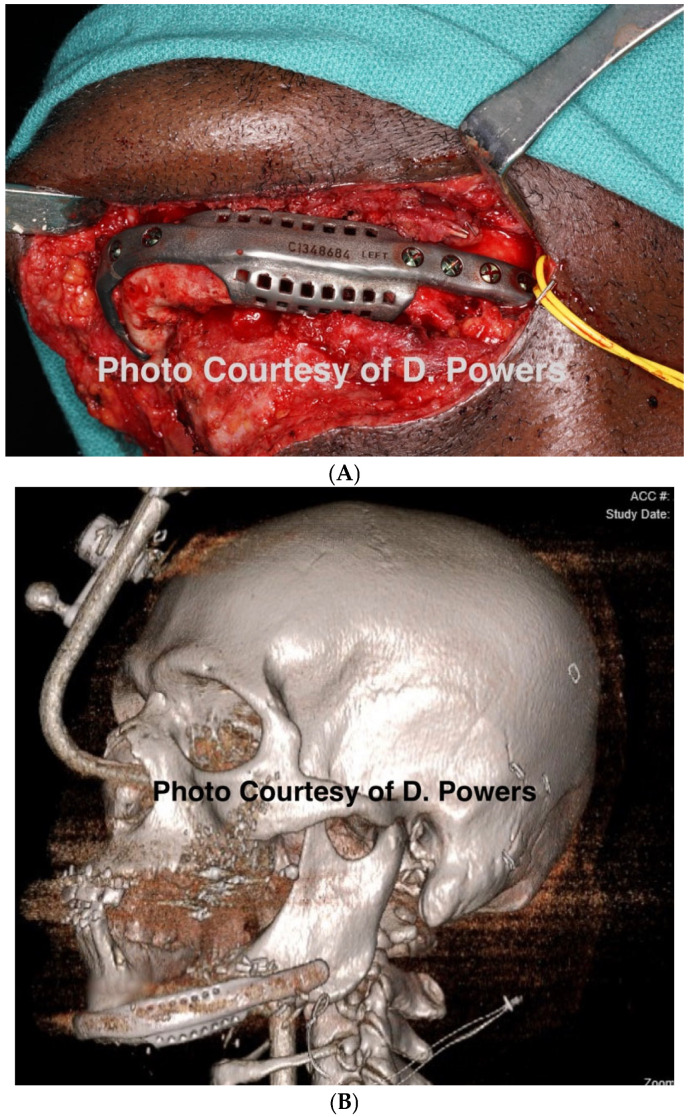
(**A**) Milled plate for mandibular reconstruction of gunshot wound. (**B**) Intraoperative computed tomography scan verifying anatomic placement.

By the mid-to-late 1980s, internal fixation had evolved from management with stainless steel wire to utilizing titanium plates and screws. This evolution has continued, and the DICOM data obtained following a contemporary CT scan can be used for the fabrication of additively manufactured custom titanium hardware with high flexural strength. Custom hardware may provide greater certainty in the spatial reconstitution of bony buttresses, but this requires additional design and manufacturing time. Although relatively costly compared with stock hardware, this cost may be offset by a reduced operating time [104] (Figure 13A,B).

Consensus Recommendation: Patient specific implants, if available, provide a near-anatomic reconstruction of the hard tissue after avulsive tissue loss. This, in turn, allows for a more natural soft-tissue reconstruction for patient aesthetics. Stereolithographic models of the patient’s deformity provide similar benefits for the adaptation of stock surgical hardware to the patient’s specific injury pattern.

#### 5.2.4. Nutrition

High-energy injury to the head and neck will frequently disrupt the functions of mastication and deglutition, if, indeed, the patient is conscious to attempt these. Increasingly recognized is the contribution of optimal nutrition to wound healing [105]. The early establishment of a feeding route (orogastric, nasogastric, percutaneous gastrostomy, or parenteral) is crucial to prevent the patient from entering a catabolic state. A Nutritional Medicine consultation will enable the tailoring of nutritional input to the patient’s specific needs.

Consensus Recommendation: In patients with ballistic craniomaxillofacial injuries, the early assessment of masticatory and swallowing function and the prompt establishment of a reliable enteral or parenteral feeding route are essential to prevent catabolism and support wound healing. Nutritional management should be initiated early through multidisciplinary collaboration, with specialist nutritional consultation to individualize caloric and protein requirements during the acute and reconstructive phases of care.

On completion of Phase II, the patient should have a clean wound bed free from necrotic or infected tissue. A high-resolution CT scan should be available to allow further study of the patient’s injuries and, if necessary, the fabrication of custom hardware. The patient should have an established route to safely meet their nutritional requirements without compromising orofacial healing. As anatomically accurate as possible, the patient’s facial buttresses, occlusion, and soft-tissue envelope dimensions should have been reconstituted with internal or external hardware, even if avulsive or segmental defects remain.

### 5.3. Phase III—Reconstruction

#### 5.3.1. Bony Reconstruction

Acceptable soft-tissue vascularity is critical for bone survival, and this, in turn, is predicated on the preceding phases, which optimize the wound bed. In the midface and upper face, where necessary, small bone gaps may be filled with non-vascularized bone grafts. Cranial bone grafts are often readily available within the operative field and have been demonstrated to have excellent survival rates, even when exposed to the sinonasal tract, as long as they are covered with healthy soft tissue [106].

In the mandible, the union of bone fragments in continuity is generally successful if they are rigidly fixated and the surrounding soft tissue can be primarily closed [12]. It can be problematic to attain reliable soft-tissue closure in the anterior mandible when there has been a loss of the genial tubercles, as the pull of the genioglossus muscle (e.g., upon swallowing) tends to cause the wound to reopen. For segmental defects, whether vascularized or non-vascularized bone grafts are used depends on the length of the defect and the quality of the surrounding tissues. Generally, mandibular defects longer than 5–6 cm may benefit from vascularized bone transfer, even when the surrounding soft tissues appear to be in good condition. If the injury has avulsed a significant area of the floor of the mouth and mandible, vascularized tissue is essential [84].

Early intervention is preferred with regard to bony reconstruction. The definitions of immediate definitive reconstruction being performed within 48 h of injury, early definitive reconstruction occurring within 30 days of injury, and delayed definitive reconstruction performed past 30 days of injury can confuse the operative surgeon regarding timing [22]. While the definition of “*early*” varies in the literature, reconstructive care should be pursued as soon as the patient is stable for surgery with *definitive* bony stabilization and soft-tissue support provided within 14 days of the injury to prevent irreversible scar contracture. As previously noted, among the selected studies for this paper reporting timing, 10 of 20 (50%) supported immediate (<48 h) and 8 of 20 (40%) supported early (<30 days) definitive intervention once physiologically feasible [21,22,23,24,25,26,31,32,33,34,35,36,37,42,43,47,49,53,54,57]. Only 2 (10%) reported delayed reconstruction, which was associated with increased soft-tissue contracture and more complex revision surgery [27,44].

Consensus Recommendation: Early definitive bony reconstruction within 10–14 days leverages the window during which the soft-tissue envelope remains extensible and regional vascularity favors graft/flap perfusion. Delays beyond this period increase scar contracture, distort facial buttresses and occlusion, and complicate both surgical access and aesthetic outcomes. Non-vascularized bone grafts are appropriate for small defects in the craniofacial skeleton when covered by healthy soft tissue, whereas mandibular defects exceeding 5–6 cm or associated with significant floor-of-mouth loss generally warrant vascularized bone transfer.

#### 5.3.2. Cranial Vault and Frontal Sinus

Ballistic injury to the frontal sinus represents a high-energy pattern frequently associated with comminution, heavy contamination, evolving tissue devitalization, and intracranial communication, requiring the modification of traditional frontal sinus fracture algorithms. While cranial vault and frontal bone reconstruction historically relied on stock titanium mesh, materials currently used for reconstruction include patient-specific PEEK and custom perforated titanium, and porous polyethylene resulting in a reduction in erosion through the overlying soft tissues by the implant. Contemporary management remains driven by posterior table involvement, frontal sinus outflow tract (FSOT) injury, and the presence of a cerebrospinal fluid (CSF) leak, but ballistic mechanisms lower the threshold for definitive sinus elimination strategies [107]. Early priorities include multidisciplinary evaluation, thin-cut CT imaging, meticulous debridement, and secure separation of the intracranial and sinonasal compartments. In cases of significant posterior table disruption, dural violation, or persistent CSF leak—common in ballistic trauma—cranialization with complete mucosal removal, FSOT obliteration, and vascularized tissue interposition are widely favored. Anterior table repair is reserved for selected cases once intracranial safety is achieved. Staged reconstruction is often appropriate given contamination and the evolving zone of injury, with long-term surveillance required to mitigate late complications such as mucocele formation and chronic infection.

Ballistic craniomaxillofacial injuries involving the anterior and middle skull base are frequently associated with traumatic brain injury, dural disruption, and cerebrospinal fluid (CSF) leak, necessitating early multidisciplinary management. High-energy mechanisms produce cavitation, contamination, and an evolving zone of injury, lowering the threshold for aggressive intracranial–sinonasal separation compared with blunt trauma. Thin-cut CT imaging is essential for identifying posterior table disruption, pneumocephalus, intracranial hemorrhage, and dural violation. A persistent CSF leak, significant posterior table comminution, or intracranial communication commonly favor cranialization with complete mucosal removal, frontal sinus outflow tract obliteration, watertight dural repair, and vascularized tissue interposition, most often with a pericranial flap. Endoscopic skull base repair may be selectively used in stable patients with localized defects, but ballistic contamination and complex fractures frequently necessitate open approaches [107]. The early control of CSF leakage reduces the risk of meningitis and intracranial infection, while staged reconstruction is often appropriate given tissue viability concerns and associated brain injury.

Consensus Recommendation: Ballistic injuries involving the cranial vault and frontal sinus should be managed using a protocol that prioritizes early multidisciplinary assessment, thin-cut CT imaging, meticulous debridement, and the secure separation of the intracranial and sinonasal compartments. Given the high incidence of posterior table disruption, dural violation, and a CSF leak in ballistic mechanisms, a lower threshold for definitive sinus elimination—most commonly, cranialization with complete mucosal removal, FSOT obliteration, watertight dural repair, and vascularized tissue interposition—is recommended, with staged reconstruction favored in the setting of contamination, evolving tissue devitalization, and associated brain injury.

#### 5.3.3. Free-Tissue Transfer

Composite free-tissue transfer may be necessary in order to reconstruct areas of both hard and soft tissue that have been avulsed or lost due to infection and necrosis. In Phase III, this should be in the context of stabilized facial buttresses and preserved overall soft-tissue envelope dimensions. Although importing distant tissue to the facial region may result in a compromised aesthetic outcome due to skin color and quality mismatches, restoring continuity and soft-tissue linings takes precedence, with local tissue held in reserve for secondary reconstruction (Phase IV).

Success in surgical free-tissue transfer has improved exponentially over the preceding decades, and the current expectation is for flap survival rates of over 95% [84]. The increased flap survivability has been a result of improvements in preoperative optimization, surgical technique, salvage procedures, and the employment of technology such as implantable Doppler probes, indocyanine-green laser-assisted fluorescence angiography, and near-infrared spectroscopy non-invasive flap oxygen saturation monitoring. As the volume and reliability of free-tissue transfer surgery have increased, the overall surgery time has been reduced, allowing surgeons to explore procedures such as innervated free flaps and concomitant dental implants [107,108,109]. Free flaps, conducted with virtually planned patient-specific osteotomies and cutting guides, are now readily available in high-volume centers, achieving near-anatomic reconstruction [110].

The complex, or avulsive, wound resulting from high-energy ballistic injury historically meant that free-tissue transfer was attempted only after complete soft-tissue healing had occurred and staged reconstructive surgery could take place in a sterile environment [111,112]. However, this cautious approach provides time for the scar contracture of the surrounding soft-tissue envelope, which irreversibly compromises the final functional and aesthetic result. With modern free-tissue transfer techniques, it has become possible to reliably import vascularized hard and soft tissue into a hostile wound environment, which permits the reconstruction of avulsed tissues before scar contracture can occur [112].

**Figure 14:**
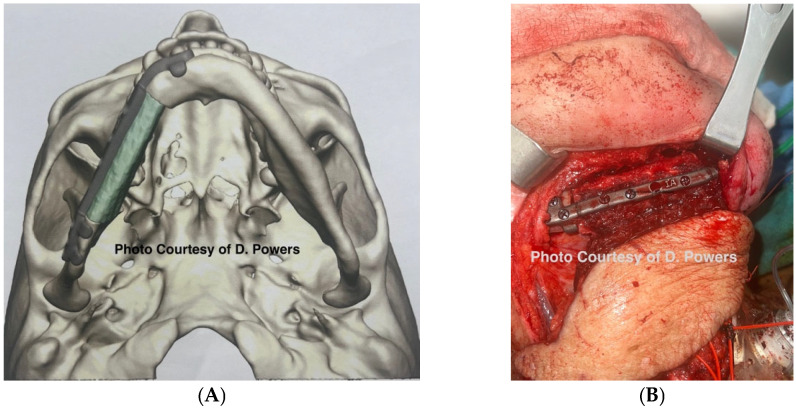
(**A**) Virtual surgical planning of mandibular reconstruction with a fibula microvascular flap. (**B**) Clinical photograph of insertion of the fibula with skin island to simultaneously reconstruct the associated soft-tissue defect.

Futran et al. reported a series of 49 patients who underwent the early definitive management of severe facial trauma utilizing free-tissue transfer [84]. Forty of the included patients had injuries resulting from gunshot wounds, twenty-six of which were self-inflicted. They note a nearly 10% rate of return to the operating room for neck exploration in this series of patients (compared with a rate of 4.2% in their wider series of 932 free flaps), and attribute this to the degree of tissue disruption, wound contamination, and inflammation resulting from the ballistic trauma [84]. Nevertheless, despite—and, perhaps, owing to—the increased take-back rate, they reported the survival of all 54 flaps used in their series. Futran et al. favored the fibula flap for mandibular reconstruction, and the scapula flap for midface defects, reserving composite radial forearm flaps for anterior maxillary defects or to augment the mandibular bone when no segmental defect was present [84] (Figure 14).

Consensus Recommendation: Composite free-tissue transfer is recommended in Phase III for ballistic craniomaxillofacial injuries with an avulsive loss of both hard and soft tissues once the facial buttresses are stabilized and the soft-tissue envelope is preserved. Modern microsurgical techniques permit reliable early definitive reconstruction in contaminated or hostile wound environments, allowing the restoration of continuity and function before scar contracture occurs, with vascularized tissue prioritized over aesthetic considerations and local tissues reserved for secondary refinement. When performed in experienced, high-volume centers, free-tissue transfer—often aided by virtual planning and patient-specific guides—achieves high flap survival rates despite the increased re-exploration risk in ballistic wounds.

#### 5.3.4. Face Transplantation

The concept of transplantation is, perhaps, the ultimate reconstructive procedure for restoring the integrity, appearance, and, potentially, function of the face following severe avulsive trauma. The first face transplant was reported in 2005 by a team in France [113]. To date, 50 partial and full-face transplants have been reported worldwide [114]. However, this treatment is not without significant technical, ethical, immunological, and psychosocial challenges, as well as a 22% long-term mortality rate from infection, malignancy, immunosuppression, non-compliance, and suicide [115]. Indications for face transplantation include extensive craniofacial defects not amenable to conventional reconstruction involving mid- and central aesthetic facial units and/or key anatomical and functional structures (e.g., orbicularis oculi and/or orbicularis oris muscles) [116]. It is self-evident that achieving success in such a procedure requires careful patient selection, highly skilled microvascular and reconstructive teams, and a well-established, multidisciplinary transplant team.

Consensus Recommendation: Facial allotransplantation should be considered a highly selective, last-resort reconstructive option for patients with devastating ballistic craniofacial injuries that are not amenable to conventional autologous reconstruction, particularly when central aesthetic units or critical functional structures are irreversibly lost. Given the substantial technical complexity, lifelong immunologic risk, psychosocial burden, and associated long-term mortality, this intervention should be reserved for carefully selected patients and performed only in specialized centers with established multidisciplinary transplant expertise and a long-term follow-up infrastructure.

#### 5.3.5. Nerve Injuries

Nerve injuries are common in ballistic craniomaxillofacial trauma and result from direct transection, cavitation, ischemia, and secondary fibrosis, often producing more severe and unpredictable deficits than blunt mechanisms. The early documentation of neurologic function and high-resolution imaging are essential, as management is guided by deficit completeness, suspected transection, anatomic location, and associated skeletal and soft-tissue injury. Suspected facial nerve transection, particularly along extratemporal trajectories involving the parotid, cheek, or temporal bone, warrants a low threshold for early exploration and repair, as the literature consistently demonstrates superior functional outcomes when tension-free neurorrhaphy or interposition grafting is performed within 72 h to 7 days, and preferably before 3 weeks, to minimize fibrosis and motor endplate degeneration [117,118]. When primary repair is not feasible, nerve transfers or dynamic reanimation are typically considered within 6–12 months to optimize recovery. Trigeminal sensory nerve injuries (infraorbital, mental, and lingual) may be observed when partial or improving; however, complete disruption or persistent neuropathic pain may justify microsurgical repair, ideally within 3–6 months, before irreversible central sensitization occurs [119]. In contrast, for optic nerve injury associated with orbital apex or skull base ballistic trauma, the current evidence does not support routine high-dose corticosteroids or optic canal decompression, with management instead focused on the urgent treatment of reversible causes such as orbital compartment syndrome and the stabilization of associated cranio-orbital injuries [120].

Consensus Recommendation: Ballistic craniomaxillofacial nerve injuries should be managed with early standardized neurologic documentation and high-resolution imaging, followed by timely, anatomy-specific intervention based on deficit severity and suspected transection. Suspected facial nerve transection warrants a low threshold for early exploration and primary repair or grafting—ideally within 72 h to 7 days (and preferably before 3 weeks)—with nerve transfers or dynamic reanimation considered within 6–12 months when primary repair is not feasible; trigeminal sensory nerve injuries may be observed if improving but should undergo microsurgical repair within 3–6 months when complete disruption or refractory neuropathic pain persists. Routine high-dose corticosteroids and optic canal decompression are not recommended for traumatic optic neuropathy; management should prioritize the urgent treatment of reversible causes, such as orbital compartment syndrome, and the stabilization of associated cranio-orbital injuries.

#### 5.3.6. Transition to Phase IV

Bony and overlying cutaneous defects should ideally be reconstructed within 10–14 days of injury to prevent wound contracture and excessive scarring [99]. This is a slightly more permissive timescale than that originally advocated by Robertson and Manson, based on the somewhat extended repatriation times contained within the recent military literature from Iraq and Afghanistan. Breeze and Bryant advocated a balanced approach in terms of the timing of definitive reconstruction, with the cleanliness of the wound bed after serial debridement balanced against the increasing potential for scar contracture with unduly delayed intervention [3].

The preceding phases of careful decontamination/debridement, accompanied by the frequent irrigation of the wound bed, followed by the reconstitution of the facial buttresses and soft tissues, and early free-tissue transfer, permit a single healing period for the injury and reconstruction. If conducted successfully, the postoperative facial proportions will be similar to the premorbid proportions, there will be an oral seal, and sufficient bone in an anatomically correct configuration will be present to allow implant placement and oral rehabilitation.

### 5.4. Phase IV—Secondary Reconstruction and Rehabilitation

Although these items are typically addressed during the final phase of injury management, the negative and positive impacts on function must be considered throughout therapy, as they represent the ultimate goals of surgical reconstruction.

#### 5.4.1. Oral Rehabilitation

A return to a functional and cosmetically acceptable dentition is often the patient’s most pressing concern following the early recovery from a devastating facial injury. Fortunately, the last quarter-century has yielded significant improvements in both the means and speed of dental rehabilitation. Limited mouth opening may be present at this stage due to a combination of the injury and treatment (e.g., prolonged maxillomandibular fixation), and therapy should be instituted as soon as the surgeon is confident that it will not compromise healing. Not only does trismus impact the patient’s quality of life, but it also limits surgical access for intraoral procedures. Powers et al.’s protocol for physical therapy in injured soldiers returning from Operation Iraqi Freedom and Enduring Freedom involved mandibular range-of-motion exercises followed by use of the TheraBite^®^ (Atos Medical, Malmö, Sweden) device in refractory cases [99]. Their TheraBite^®^ protocol consisted of seven mandibular stretches, each held at the maximum opening for seven seconds, repeated seven times per day (7-7-7 protocol), until the patient achieved a maximum interincisal opening greater than 35 mm or no further improvement was observed. For this to be successful, a diligent and compliant patient is required. If recalcitrant temporalis muscle fibrosis is present, coronoidectomies followed by further brisement procedures are indicated.

**Figure 15:**
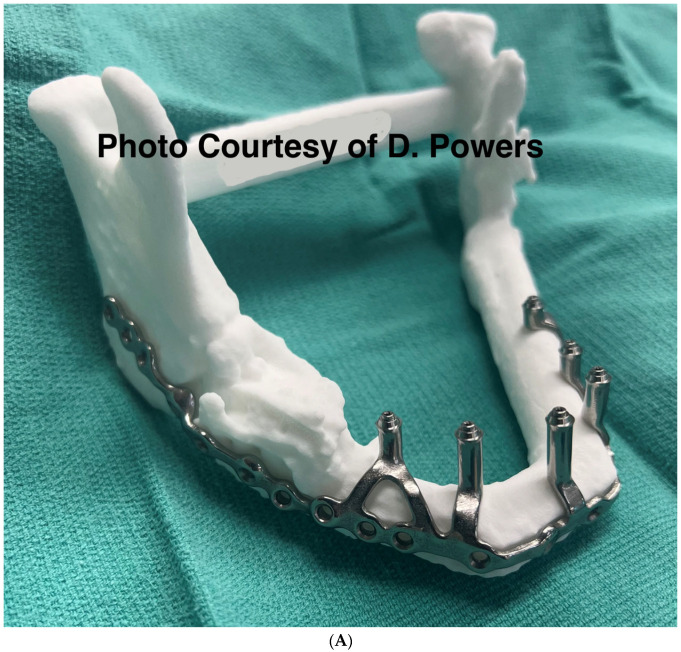

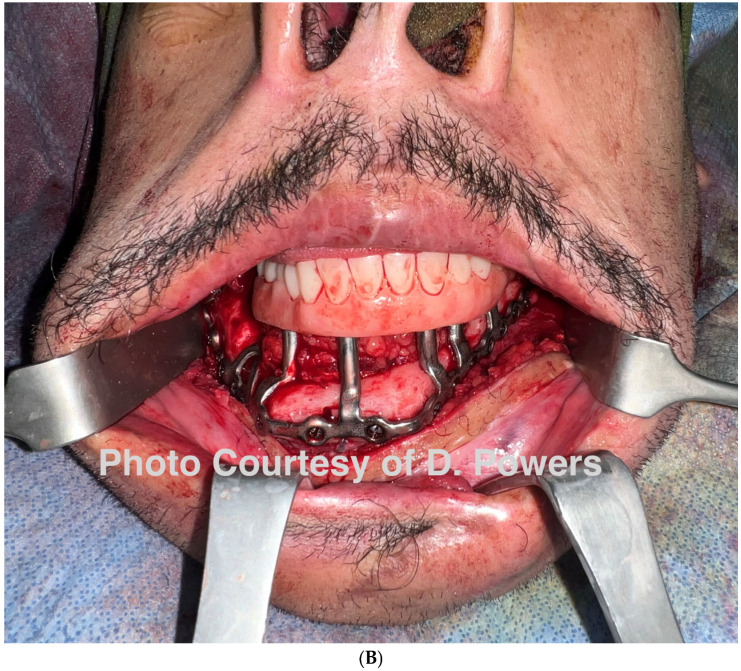
(**A**) Virtual model of a patient who lost the majority of his mandible secondary to an explosive blast. The osseous reconstruction was accomplished with a fibula osteocutaneous graft and the dental rehabilitation was planned with a patient-specific bone-anchored subperiosteal implant. (**B**) Clinical photograph of the insertion of the prosthesis with the attached mandibular denture.

If implants are not already present in the allotransplanted tissue, all-on-four protocols have been developed to allow so-called *“Jaw in a Day”* reconstruction. If there is insufficient bone for conventional dental implants, zygomatic and pterygoid implants may be an alternative. Suppose the available bone is entirely unsuitable for endosseous implants. In that case, a recent advance is the additively manufactured patient-specific bone-anchored subperiosteal implant, which has provided new hope for oral rehabilitation in patients with bone depletion [121,122] (Figure 15A,B).

Consensus Recommendation: Oral rehabilitation should be initiated early once skeletal and soft-tissue healing permit, as the restoration of functional dentition is central to quality of life following ballistic craniomaxillofacial injury. The early institution of mandibular range-of-motion therapy is recommended to address trismus and facilitate subsequent intraoral access, with an escalation to adjunctive devices or surgical release when conservative measures fail. Contemporary implant-based strategies—including immediate full-arch protocols, alternative anchorage such as zygomatic or pterygoid implants, and patient-specific subperiosteal implants—should be considered within a multidisciplinary framework to achieve timely, stable, and functional dental rehabilitation.

#### 5.4.2. Aesthetic Management

Once the objectives of Phase III have been achieved, the secondary revision of bulky or color-mismatched cutaneous tissue can be performed. There are several options for this, but locoregional tissue rearrangement often provides the best skin color and quality match. Menick, for instance, routinely revises his free-tissue transfer nasal reconstructions with a forehead flap [123]. The cutaneous portion of a color-mismatched free flap can be secondarily excised and replaced with local tissue to optimize aesthetics, or an alternative option is cosmetic tattooing, in order to more closely simulate the shade of the adjacent tissue [124,125,126].

Suppose avulsive injuries to the ears or nose have not been amenable to local or distant tissue reconstructive procedures. In such cases, excellent aesthetic results can be achieved with the input of a skilled maxillofacial prosthetist and an implant-retained prosthesis. For tissue volume deficiencies, patient-specific onlays can now be fabricated in a range of materials (e.g., porous polyethylene, polyether ether ketone (PEEK), and titanium), which can be employed as an adjunct to symmetrize the volume as needed. The use of high-resolution DICOM data in the fabrication of these can be employed to mirror the uninjured side onto the injured side and construct an implant to compensate for the difference [127]. If the volume deficiency is primarily due to a lack of soft rather than hard tissue, fat transfer may be beneficial in ameliorating this deficit, as well as improving the quality of the overlying skin [128,129].

The dermabrasion of scars 4–6 weeks after soft-tissue closure or scar revision is beneficial in levelling the skin and improving cosmesis [99]. Intradermal triamcinolone is effective in reducing hypertrophic scarring, as is topical silicone sheeting [130,131]. Scarring resulting from ballistic and blast injuries is frequently associated with the adverse tattooing of the skin due to penetration with small metallic fragments. Gray-blue pigmentation may be treated using the pulsed dye laser (595 nm, a yellow light laser) on a monthly basis, with 8–10 applications. The Q-switched laser may also be effective in disrupting fragments of copper, zinc, graphite, and other metals through photo-acoustic shattering. Powers et al. also recommend the Ng-YAG (1064 nm) infrared laser and the red Alexandrite laser (755 nm) as beneficial for reducing adverse tattooing [99].

As part of the Phase IV aesthetic optimization, the patient should be referred to a professional cosmetologist and counseled regarding hairstyle, makeup, and eyewear in order to enhance the positive aspects and camouflage the negative aspects of the final reconstruction [84].

Consensus Recommendation: Aesthetic optimization following ballistic craniomaxillofacial reconstruction should be pursued in a staged, secondary manner once functional stability is achieved, prioritizing locoregional tissue rearrangement to improve skin color, texture, and contour match. Adjunctive strategies—including patient-specific onlays, fat transfer, prosthetic rehabilitation, scar modulation, laser therapy for traumatic tattooing, and cosmetologic support—play an essential role in refining outcomes, managing disfigurement, and enhancing patient confidence and social reintegration during Phase IV recovery.

#### 5.4.3. Psychological Management

Facial appearance is closely tied to identity, self-perception, and attractiveness. Faces play a central role in human interaction, first impressions, non-verbal communication, and assumptions about personality. Every ballistic injury should be considered life-changing—in terms of function, appearance, and psychological impact. In a US study of fatal civilian firearm injuries, 62% were suicides, 35% homicides, and 2% unintentional firearm deaths. In contrast, for non-fatal civilian firearm injuries, 72% were related to assault, 17% were unintentional, and 5% were related to self-harm [132]. Actualized self-harm implies a pre-existing psycho-morbidity, while being a victim of an assault or unintentional firearm injury exacts a psychological toll. Bisson et al. found 12/43 (27%) of all facial trauma patients (including non-ballistic) to be suffering from post-traumatic stress disorder (PTSD) 7 weeks after their facial injury; this was corroborated by Lal et al. who showed that symptoms persisted in 10% at 6–12 months post-injury [133,134]. Other reviews point toward a significantly increased prevalence of PTSD, anxiety, and depression in facial trauma patients, particularly in victims of assault [135,136].

Tsur et al. examined a military cohort with traumatic maxillofacial injuries sustained in non-military and military circumstances [137]. They found military maxillofacial trauma (gunshot and shrapnel injuries) to be associated with a significantly higher rate of PTSD (30%) compared with trauma sustained in non-military circumstances (14%). This finding corroborated the results from studies in other anatomical regions that report a correlation of PTSD with military injuries and injury severity [138,139].

The stigma of disfigurement can cause difficulties in maintaining self-esteem, building self-confidence, and coping with the unsolicited negative reactions of others [136]. Changes to the face are particularly noticeable to the affected person and to observers, with symmetrical, ‘average’ faces (those with less extreme characteristics) widely perceived as the most attractive, and those individuals being treated as more popular, brighter, kinder, more socially skilled, more likeable, and morally better than less attractive individuals [140,141]. In an experiment using retouched photographs, people with abnormal faces (due to congenital deformity or post-traumatic scars) were rated as significantly less honest, less employable, less trustworthy, less optimistic, less effective, less capable, less intelligent, less popular, and less attractive than the same people with atraumatic/non-pathologic facial appearances, irrespective of the evaluator’s educational level, sex, or age [142].

The importance of a good aesthetic outcome following facial trauma is, hence, difficult to overstate. Attaining such an outcome, however, particularly after injuries involving avulsion of tissues, is a significant challenge and almost invariably requires multiple procedures. Futran et al. reported an average of eight surgical procedures being required when nasal tissues were involved in the injury [84]. The patient’s and family/friends’ expectations should be managed early on in this regard, with the senior authors’ (P.M. and D.P.) standard practice being to clearly articulate that this will be a life-changing injury, ultimately requiring between 8–12 surgical procedures over the course of many months, or years, to reach the final result.

Consensus Recommendation: Ballistic craniomaxillofacial injuries should be managed with early and ongoing psychological assessment and support, recognizing that these injuries are inherently life-altering and carry a high risk of PTSD, anxiety, depression, and psychosocial impairment, particularly in assault- and combat-related contexts. Psychological care—including early mental health consultation, screening for self-harm risk, and longitudinal support—should be integrated alongside surgical management, with the clear, realistic counseling of patients and families regarding the prolonged reconstructive course and anticipated functional and aesthetic outcomes to optimize long-term recovery and quality of life.

#### 5.4.4. Other Considerations

##### Hyperbaric Oxygen Therapy (HBOT)

The zone of injury in a high-energy ballistic wound may be attenuated by HBOT, reoxygenating the microcirculation. While not a new therapy, evidence in the literature remains limited and indirect [143,144]. Although it is possible to treat patients with HBOT in dedicated facilities, in most locations, this is logistically impossible.

##### Retained Fragments

Fragments from bullets are often retained after injury, as dissecting out each fragment usually carries more immediate risk than leaving them in situ. Complications arising from retained fragments depend, in large part, on their location, but include infection, spontaneous migration, embolism, arthritis, and lead toxicity [145]. Most general indications for prophylactic removal—bullets that are intra-articular, intra-bursal, or on a weight-bearing surface such as the palm or sole of the foot—do not apply to the head and neck [146]. Retained fragments located in soft tissues become enclosed by avascular scar tissue, reducing the risk of infection and heavy metal absorption. Lead is highly soluble in synovial fluid, however, and a bullet retained in an articular space can result in both local and systemic effects. Lead poisoning (plumbism or saturnism) is a rare, but reported, occurrence from fragments retained in the head and neck [146,147]. However, if a pseudocyst forms around the bullet fragments rather than scar tissue, this may permit the release of lead by macrocytic phagocytosis, which can then be absorbed into the bloodstream [148].

Lead toxicity can be difficult to diagnose due to its vague symptoms and insidious onset. The Centers for Disease Control and Prevention halved the threshold for an elevated venous blood lead level (BLL) in adults to 5 μg/dL [149]. Symptoms typically occur at a BLL of >24 μg/dL, and include headache, fatigue, nausea, abdominal pain, constipation, forgetfulness, irritability, depression, and mood change [148,149]. Toxicity is positively correlated with the number of retained fragments, the presence of bony fractures, the duration of bullet retention, and the formation of pseudocysts [148].

In recent years, there has been an increasing call for BLL monitoring in gunshot wound patients with retained bullets or fragments. A suggested protocol is monitoring at 3-month intervals for the first year. If the BLL is elevated (according to the CDC’s criteria), the removal of the retained fragment is recommended, as this has been shown to reduce BLLs even decades after the GSW incident [150,151]. Morbidity associated with access for removal can be reduced by utilizing the aforementioned technological adjuncts, including up-to-date cross-sectional imaging and surgical navigation systems, to locate fragments and foreign bodies precisely. If there is concern about BLLs being acutely elevated during the procedure, chelation therapy can be initiated.

Consensus Recommendation: Retained ballistic fragments in the craniomaxillofacial region should generally be left in situ unless they pose a specific risk, as routine removal often carries greater morbidity than observation. Indications for intervention include fragment-related infection, migration, neurovascular compromise, symptomatic pseudocyst formation, or evidence of lead toxicity, with serial blood lead level monitoring recommended—particularly in patients with multiple retained fragments or associated bony injury. When removal is indicated, contemporary imaging and intra-operative navigation techniques should be employed to minimize surgical morbidity, with chelation therapy considered in cases of significant or rising lead burden.

### 5.5. Complications

In the complex, contaminated wounds resulting from high-energy ballistic trauma, complications should be regarded as an expected—though undesirable—component of treatment rather than a failure of care. Reported complication rates vary widely according to the mechanism, energy transfer, and wound location. Peled et al. described a 22% complication rate (wound infection and flap dehiscence) in Israeli soldiers with high-energy facial injuries [152]. Kihtir et al. reported wound-healing complications in 39% of patients with low-energy gunshot wounds traversing the oral cavity [153], while close-range shotgun injuries to the face have been associated with infection rates approaching 100% [154]. These figures underscore the hostile biologic environment in which reconstruction must occur and highlight that even meticulous technique cannot completely eliminate adverse events.

In response to these challenges, Shvyrkov advocated radical primary surgical debridement (RPSD) during the Russian–Afghanistan War, reporting a tenfold reduction in soft-tissue infection from a previously documented 47.6% complication rate [155]. However, such aggressive debridement risks the excessive loss of viable facial soft tissue, forcing compromises in projection and contour for primary closure or necessitating large rotational or microvascular flaps that may adversely affect both function and aesthetics. For this reason, neither of the senior authors (P.M. and D.P.) has adopted RPSD for ballistic injuries, and this approach is not reflected in the recent United States Military Clinical Practice Guidelines or in the experience of Peled et al. with Israeli casualties [152,156,157].

Consistent with Robertson and Manson’s original counsel, wound infection, dehiscence, or localized breakdown should be viewed as manageable setbacks rather than catastrophic failures that threaten the long-term outcome [1]. Within the framework of this four-phase protocol, careful staged debridement, the preservation of potentially viable tissue, the maintenance of facial buttresses and soft-tissue envelope dimensions, and early definitive reconstruction are prioritized. This philosophy accepts a certain rate of short-term wound complications in exchange for maximizing the patient’s ultimate functional and aesthetic result.

#### Future Directions and Research Priorities

To advance the evidence-based management of ballistic craniomaxillofacial trauma, priority areas include the following:

- A multicenter civilian registry development with standardized outcome reporting, with national military collaboration to this registry;
- A comparative evaluation of immediate versus delayed free-flap reconstruction;
- The validation of civilian adaptations of <C>ABC protocols;
- A high-resolution quantification of soft-tissue envelope dynamics after injury;
- Outcomes-based timing studies for airway, nutrition, and navigation-guided debridement.

Robust prospective data are needed to refine timing guidance and quantify the long-term functional and psychosocial impact of early versus delayed reconstruction. It is imperative for both the civilian and military communities to facilitate this data acquisition and honestly share the functional and aesthetic outcomes of their treatment.

## 6. Study Limitations and Risk of Bias

The overall quality of the evidence was low to moderate. Most studies were single-center, retrospective cohorts or case series with heterogeneous patient populations, mechanisms of injury, and reconstructive resources. The timing of intervention and definitions of complications were inconsistently reported, and the follow-up duration varied widely. These factors introduce selection and reporting biases and limit the direct comparison across studies.

Consequently, the recommendations presented in this protocol reflect a synthesis of the available evidence and expert consensus derived from high-volume civilian trauma centers and battlefield experience. The expert consensus was developed through an iterative review of contemporary literature, historical war surgery doctrine, and collective clinical experience managing ballistic craniomaxillofacial injuries across diverse care environments. Areas of agreement were identified by comparing independently reported management strategies and outcomes, with emphasis placed on principles and interventions that demonstrated a consistent application across multiple institutions and practice settings. Practices supported only by isolated experience or lacking reproducibility were intentionally de-emphasized. Given the ethical and logistical constraints that limit high-level comparative trials in ballistic trauma, this consensus-based approach provides pragmatic, experience-informed guidance while acknowledging the need for ongoing refinement as higher-quality data emerge. Where evidence was limited, the recommendations were anchored to damage-control principles, anatomic reconstruction priorities, and risk mitigation strategies that have demonstrated durability across both civilian and military trauma systems.

## 7. Conclusions

Hippocrates famously commented that *“War is the only proper school for a surgeon”*, and, since the publication of Robertson and Manson’s original paper, there have been a range of surgical advances directly attributable to battlefield experiences in the last quarter century, as well as one of the senior author’s own personal knowledge of combat surgical procedures in an austere environment during his prior military career (D.P) [158]. Although technology and surgical success rates have improved significantly since 1999, the central tenets of Robertson and Manson’s protocol remain essentially unchanged. The excision of necrotic tissue followed by the serial debridement and repair of the bony buttresses of the face remains the foundation on which successful further reconstruction depends [1].

What has evolved most strikingly is the manner in which these foundational steps are now executed. The widespread availability of intraoperative imaging—including portable CT, cone-beam CT, and image-guided navigation—permits the real-time verification of anatomic reduction, the retrieval of retained ballistic fragments, and the confirmation of hardware placement. This technology reduces intraoperative uncertainty, decreases the likelihood of secondary revisions, and improves the accuracy of skeletal reconstruction [159,160].

In parallel, patient-specific implants (PSIs) produced via virtual surgical planning and additive manufacturing have transformed the reconstructive paradigm. Ballistic injuries are often characterized by irregular comminution and tissue loss that make stock implants inadequate. With PSIs, implants can be precisely designed using pre-injury imaging or contralateral templates, thereby restoring buttress integrity, orbital volume, and mandibular continuity with high accuracy [159,160]. When combined with intraoperative imaging, PSIs have shortened operative times, optimized functional outcomes, and improved aesthetic restoration [161,162]. These advancements are summarized in a recommended treatment flow chart, identified as Table 2.

Although many contemporary advances in ballistic craniomaxillofacial trauma management rely on resource-intensive technologies, several high-impact interventions have demonstrated effectiveness across lower-funded care environments. Large multicenter trauma trials evaluating early tranexamic acid administration illustrate how inexpensive, protocol-driven interventions can significantly reduce mortality, reinforcing the primacy of physiology-based damage-control principles over technology dependence on hemostatic dressing agents [163]. Foundational surgical practices—including early airway protection, hemorrhage control, meticulous debridement, infection prevention, and staged reconstruction—remain the primary determinants of outcome in ballistic injuries. Advanced strategies such as virtual surgical planning, navigation, and patient-specific implants may be selectively adopted in low-resource regions through tiered, regionalized models using open-source planning platforms and the low-cost three-dimensional modeling or utilization of stock facial skeletal models as templates for anatomically designing stock reconstructive plates. Standardized definitions and outcome reporting further support reproducibility and incremental adoption as resources evolve.

**Figure 16:**
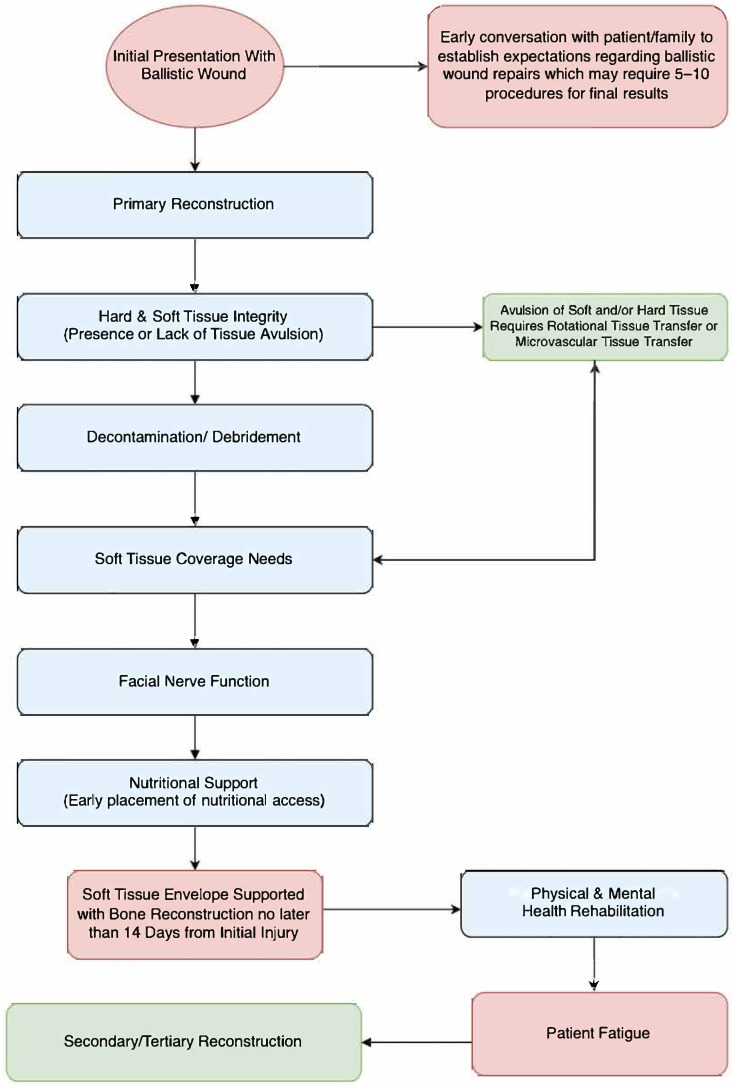
Recommended treatment algorithm for ballistic injury management.

Together, these innovations represent a natural extension of Robertson and Manson’s principles into the modern surgical era: maintaining the primacy of debridement and facial bony buttress repair while executing these steps with unprecedented precision. As such, the management of ballistic injuries to the head and neck now reflects both the enduring lessons of history and the transformative potential of contemporary surgical technology [Figure 16].

## Figures and Tables

**Table 1:** Modern Protocol vs. Robertson & Manson Original Guidance.

Domain	Robertson & Manson (Historical Standard)	Current Updated Protocol (This Paper)	Evolutionary Rationale
**Timing of Reconstruction**	Emphasized early definitive repair, but in practice, delays common due to evacuation + wound care limitations	Consistently supports early reconstruction (<10–14 days) when physiologically stable	Modern systems shorten evacuation → reduce contracture + complexity
**Phases of Care**	3-Phase approach: (1) Stabilization, (2) Reconstruction, (3) Revisions	4-Phase model: (I) Damage control, (II) Reconstitution, (III) Definitive reconstruction, (IV) Rehabilitation	Reorganization mirrors contemporary trauma surgery: <C>ABC, damage control resuscitation, soft-tissue envelope preservation
**Soft-Tissue Management**	Debridement emphasized, but limited by imaging + vascular assessment	Serial debridement + navigation-guided “zone-of-injury” definition, protect viable envelope	Better imaging = less unnecessary tissue sacrifice
**Hard-Tissue Stabilization**	Traditional hardware; staged grafting commonly required	External fixation optimized, then rigid fixation with modern precision implants	Technology → faster definitive stabilization with fewer secondary surgeries
**Use of Free Tissue Transfer**	Often delayed (weeks–months) to reduce failure risk	Early free flaps are safe and preferred → reduce contracture and revisions	Flap survival now >95% in high-energy trauma
**Navigation & Virtual Planning**	Not available	Widespread CT, 3D photogrammetry, additive-manufactured implants, navigation	Allows accurate buttress reconstruction even in avulsive injuries
**Airway Management**	Surgical airway recognized but not heavily emphasized in algorithm	Surgical airway early in anterior mandibular injuries; combat airway data incorporated	Combat evidence → <C>ABC / MARCH prioritization
**Nutrition Strategy**	Recognized but not highlighted as early determinant of success	Aggressive early nutrition + enteral access as Phase I/II priority	Catabolism delays wound healing → worsens outcomes
**Setting Differences**	Primarily civilian environment	Dual-path algorithm: civilian vs. battlefield	Recognizes differences in EMS, resource availability, evacuation
**Psychological Outcomes**	Rarely addressed in protocol	Routine mental health surveillance introduced	PTSD and appearance-related distress known and high prevalence
**Patient Expectations**	Function and closure prioritized	Appearance, identity, and quality of life explicitly included	Survivorship → long-term quality of life primary driver
**Ethics & Transparency**	Not routinely stated	Explicit image publication consent	Modern research standards & editorial guidance

**Table 2:** Summary of Evidence and Consensus Recommendations by Phase of Management.

Phase	Evidence Available (Level)	Representative Quantitative Findings	Consensus Recommendations	Civilian vs. Battlefield Variations
**I. Resuscitation** **& Damage Control**	III–IV	Tracheostomy required in ~19% of combat maxillofacial trauma; TIC in 25% major trauma	<C>ABC prioritization; whole blood when feasible	Battlefield: early whole blood; Civilian: EMS variability
**II. Stabilization & Reconstitution**	III–IV	External fixation useful for avulsive mandible; navigation reduces complications	Preserve bone/soft tissue envelope; sequential debridement	Austere settings may require prolonged temporization
**III. Reconstruction**	II–III	Free-flap survival >95%; early free flap reduces contracture	Definitive bony stabilization <10–14 days if stable	Battlefield delays often due to evacuation timelines
**IV. Secondary Reconstruction & Rehab**	III–IV	Multiple revisions typically required; ~27% PTSD early	Esthetic revision, oral rehab, psychosocial support	Higher PTSD prevalence with military trauma

## Data Availability

All of the data compilation is included in the manuscript.

## Supplementary Materials

The following supporting information can be downloaded at: https://www.mdpi.com/article/10.3390/cmtr19010014/s1, Table S1: Summary of included literature reviewed for the fabrication of this manuscript.

## References

[1] Robertson B.C., Manson P.N. (1999). High-energy ballistic and avulsive injuries: A management protocol for the next millennium. Surg. Clin. N. Am..

[2] Breeze J., Gibbons A.J., Shief C., Banfield G., Bryant D.G., Midwinter M.J. (2010). Head face and neck injuries sustained by British servicemen in Iraq and Afghanistan: 01 March 2003–31 December 2008. Br. J. Oral Maxillofac. Surg..

[3] Breeze J., Bryant D. (2009). Current Concepts in the Epidemiology and Management of Battlefield Head, Face and Neck trauma. BMJ Mil. Health.

[4] Xydakis M.S., Fravell M.D., Nasser K.E., Casler J.D. (2005). Analysis of battlefield head and neck injuries in Iraq and Afghanistan. Otolaryngol.-Head. Neck Surg..

[5] Sinyuk M., Polishchuk V., Yuschak P., Burachok I. (2025). Management of war-related facial wounds in Ukraine: The Lviv military hospital experience. BMJ Mil. Health.

[6] Breeze J., Horsfall I., Hepper A., Clasper J. (2011). Face, neck, and eye protection: Adapting body armour to counter the changing patterns of injuries on the battlefield. Br. J. Oral Maxillofac. Surg..

[7] Kosashvili Y., Hiss J., Davidovic N., Lin G., Kalmovic B., Melamed E., Levy Y., Blumenfeld A. (2005). Influence of personal armor on distribution of entry wounds: Lessons learned from urban-setting warfare fatalities. J. Trauma-Inj. Infect. Crit. Care.

[8] Khoschnau S., Ramzee A.F., El-Menyar A., Peralta R., Consunji R., Kloub A., Ajaj A., Abdelrahman H., Al-Thani H. (2023). Airbag-related penetrating injuries: A case series from a level 1 trauma center. Trauma Case Rep..

[9] Pepper T., Karia R., Radia R., Stenhouse P. (2022). 101. Orbital Foreign Body Injuries—A management algorithm and classification proposal. Br. J. Oral Maxillofac. Surg..

[10] Thomas M.D., Siu K. (1987). An unusual cranial injury caused by an industrial nail-gun. Med. J. Aust..

[11] Shadid O., Simpson M., Sizer J. (2008). Penetrating injury of the maxillofacial region with an arrow: An unsuccessful attempt of suicide. Br. J. Oral Maxillofac. Surg..

[12] Pepper T., Powers D. (2025). Open Versus Closed Management for Gunshot Wounds of the Mandible—Which More Frequently Achieves Satisfactory Bony Union? A Systematic Review and Meta-Analysis. J. Oral Maxillofac. Surg..

[13] Powers D.B., Delo R.I. (2013). Characteristics of ballistic and blast injuries. Atlas Oral Maxillofac. Surg. Clin. N. Am..

[14] Stefanopoulos P.K., Filippakis K., Soupiou O.T., Pazarakiotis V.C. (2014). Wound ballistics of firearm-related injuries-Part 1: Missile characteristics and mechanisms of soft tissue wounding. Int. J. Oral Maxillofac. Surg..

[15] Santucci R.A., Chang Y.J. (2004). Ballistics for physicians: Myths about wound ballistics and gunshot injuries. J. Urol..

[16] Ziervogel J.F. (1979). A study of the muscle damage caused by the 7.62 NATO rifle. Acta Chir. Scand. Suppl..

[17] Barach E., Tomlanovich M., Nowak R. (1986). Ballistics: A pathophysiologic examination of the wounding mechanisms of firearms: Part I. J. Trauma-Inj. Infect. Crit. Care.

[18] Fackler M.L. (1988). Wound Ballistics: A Review of Common Misconceptions. JAMA.

[19] Fackler M.L., Bellamy R.F., Malinowski J.A. (1988). The wound profile: Illustration of the missile-tissue interaction. J. Trauma-Inj. Infect. Crit. Care.

[20] Fackler M.L. (1998). Civilian gunshot wounds and ballistics: Dispelling the myths. Emerg. Med. Clin. N. Am..

[21] McGowan J., Sampson M., Salzwedel D.M., Cogo E., Foerster V., Lefebvre C. (2016). PRESS Peer Review of Electronic Search Strategies: 2015 Guideline Statement. J. Clin. Epidemiol..

[22] Vaca E.E., Bellamy J.L., Sinno S., Rodriguez E.D. (2018). Management of High-energy Avulsive Ballistic Facial Injury: A Review of the Literature and Algorithmic Approach. Plast. Reconstr. Surg. Glob. Open.

[23] Riyadh S., Abdulrazaq S.S., Zirjawi A.M.S. (2018). Surgical management of the recent orbital war injury. J. Craniofac. Surg..

[24] Eser C., Gencel E., Kesiktaş E., Yavuz M. (2016). Outcomes of anatomic reconstruction of gunshot-inflicted lower face defects by free osteoseptocutaneous fibula flap and expanded or nonexpanded temporal scalp flap combination in males. J. Craniofac. Surg..

[25] Uçak M. (2020). Alternative methods to local flap applications in large tissue losses caused by gunshot injuries in the Syrian War. J. Craniofac. Surg..

[26] Motamedi M.H.K. (2011). Management of firearm injuries to the facial skeleton: Outcomes from early primary intervention. J. Emerg. Trauma Shock..

[27] Guerrier G., Alaqeeli A., Al Jawadi A., Foote N., Baron E., Albustanji A. (2015). Reconstruction of residual mandibular defects by iliac crest bone graft in war-wounded Iraqi civilians, 2006–2011. Br. J. Oral Maxillofac. Surg..

[28] Tahmasebi E., Tabrizi R., Yazdanian M., Shafiei S., Moslemi H., Motamedi M.H.K., Mozaffari F., Torabizadeh A. (2024). Assessment of predicting success factors of microvascular composite fibula flap in reconstruction of hard and soft tissue defects in maxillofacial and oral cavity due to previous ballistic trauma. J. Popul. Ther. Clin. Pharmacol..

[29] Kummoona R., Muna A.M. (2006). Evaluation of immediate phase of management of missile injuries affecting maxillofacial region in iraq. J. Craniofac. Surg..

[30] Sadda R.S. (2003). Maxillofacial war injuries during the Iraq–Iran War. Int. J. Oral Maxillofac. Surg..

[31] Peleg M., Sawatari Y. (2010). Management of gunshot wounds to the mandible. J. Craniofac. Surg..

[32] Gurunluoglu R., Gatherwright J. (2019). Microsurgical reconstruction of complex maxillofacial gunshot wounds: Outcomes analysis and algorithm. Microsurgery.

[33] Shackford S.R., Kahl J.E., Calvo R.Y., Kozar R.A., Haugen C.E., Kaups K.L., Willey M., Tibbs B.M., Mutto S.M., Rizzo A.G. (2014). Gunshot wounds and blast injuries to the face are associated with significant morbidity and mortality: Results of an 11-year multi-institutional study of 720 patients. J. Trauma Acute Care Surg..

[34] Chaiyasate K.M., Gupta R.B., Boudiab E.M., Vega D., Hart J.D., Nossoni F.D., Lu S., Powers J.M., Hobson G.D., Sachanandani N.S. (2022). Comprehensive treatment and reconstructive algorithm for functional restoration after ballistic facial injury. Plast. Reconstr. Surg. Glob. Open.

[35] Soto E., Ovaitt A.K., Clark A.R., Tindal R.R., Chiasson K.F., Aryanpour Z., Ananthasekar S., Grant J.H., Myers R.P. (2021). Reconstructive management of gunshot wounds to the frontal sinus: An urban trauma center’s perspective. Ann. Plast. Surg..

[36] Heffern E., Nevil C., Przylecki W., Andrews B.T. (2021). Anatomic subunit approach to composite reconstruction of facial gunshot wounds. J. Craniofac. Surg..

[37] Orthopoulos G., Sideris A., Velmahos E., Troulis M. (2013). Gunshot wounds to the face: Emergency interventions and outcomes. World J. Surg..

[38] Newlands S.D., Samudrala S., Katzenmeyer W.K. (2003). Surgical treatment of gunshot injuries to the mandible. Otolaryngol.-Head. Neck Surg..

[39] Hoppe I.C., Kordahi A.M., Paik A.M., Lee E.S., Granick M.S. (2014). Pediatric facial fractures as a result of gunshot injuries. J. Craniofac. Surg..

[40] Hollier L., Grantcharova E.P., Kattash M. (2001). Facial gunshot wounds: A 4-year experience. J. Oral Maxillofac. Surg..

[41] Pereira C., Boyd J.B., Dickenson B., Putnam B. (2012). Gunshot wounds to the face. Ann. Plast. Surg..

[42] Norris O., Mehra P., Salama A. (2015). Maxillofacial Gunshot Injuries at an Urban Level I Trauma Center—10-Year Analysis. J. Oral Maxillofac. Surg..

[43] Firat C., Geyik Y. (2013). Surgical modalities in gunshot wounds of the face. J. Craniofac. Surg..

[44] Vayvada H., Menderes A., Yilmaz M., Mola F., Kızılkaya A., Atabey A. (2005). Management of Close-Range, High-Energy Shotgun and Rifle Wounds to the Face. J. Craniofac. Surg..

[45] Ucak M. (2019). Incidence and severity of maxillofacial injuries during the Syrian Civil War in Syrian soldiers and civilians. J. Craniofac. Surg..

[46] Tabakan I., Eser C., Gencel E., Kokaçya Ö. (2021). Reconstruction of firearm and blast injuries in Syrian war refugees. Int. J. Clin. Pract..

[47] Channar K., Safia Warriach A.Q. (2011). Comparison of Open Reduction and Internal Fixation Versus Closed Reduction and Maxillomandibular Fixation for the Treatment of Gunshot Injuries of Mandible. J. Liaquat. Uni. Med. Health Sci..

[48] Siddiqui S.U.D., Iqbal N., Baig M.H., Mehdi H., Mahmood Haider S. (2020). Efficacy of open reduction and internal fixation in achieving bony union of comminuted mandibular fractures caused by civilian gunshot injuries. Surgeon.

[49] Rana M., Warraich R., Rashad A., von See C., Channar K.A., Rana M., Stoetzer M., Gellrich N.-C. (2014). Management of comminuted but continuous mandible defects after gunshot injuries. Injury.

[50] Muddassar M., Arshad R., Rabbani S., Qureshi I.S., Khattak I.K., Rana Z. (2020). Management of gunshot injuries of mandible with open reduction and internal fixation versus closed reduction and maxillo-mandibular fixation. Cureus.

[51] Ahmed W., Adil Asim M., Ehsan A., Abbas Q. (2018). Non-vascularized Autogenous Bone Grafts for Reconstruction of Maxillofacial Osseous Defects. J. Coll. Physicians Surg. Pak..

[52] Bukhari S., Khan S.G., Pasha I., Ahmad B. (2010). Management of facial gunshot wounds. J. Coll. Physicians Surg. Pak..

[53] Jose A., Arya S., Nagori S. (2019). High-velocity ballistic injuries inflicted to the maxillofacial region. J. Craniofac. Surg..

[54] Jeyaraj P., Chakranarayan A. (2018). Treatment strategies in the management of maxillofacial ballistic injuries in low-intensity conflict scenarios. J. Maxillofac. Oral. Surg..

[55] Gröbe A., Weber C., Schmelzle R., Heiland M., Klatt J., Pohlenz P. (2009). The use of navigation ({BrainLAB} Vector vision(2)) and intraoperative {3D} imaging system (Siemens Arcadis Orbic 3D) in the treatment of gunshot wounds of the maxillofacial region. Oral Maxillofac. Surg..

[56] Xing L., Duan Y., Zhu F., Shen M., Jia T., Liu L., Tao J., Chen Y., Gao Z., Zhang H. (2015). Computed tomography navigation combined with endoscope guidance for the removal of projectiles in the maxillofacial area: A study of 24 patients. Int. J. Oral Maxillofac. Surg..

[57] Wei L.F., Wang S.S., Jing J.J., Zheng Z.C., Gao J.X., Liu Z., Wang R.M. (2013). Surgical therapy for craniocerebral firearm injury. Turk. Neurosurg..

[58] Holcomb J.B., McMullin N.R., Pearse L., Caruso J., Wade C.E., Oetjen-Gerdes L., Champion H.R., Lawnick M., Farr W., Rodriguez S. (2007). Causes of death in U.S. Special Operations Forces in the global war on terrorism: 2001–2004. Ann. Surg..

[59] Eastridge B.J., Hardin M., Cantrell J., Oetjen-Gerdes L., Zubko T., Mallak C., Wade C.E., Simmons J., Mace J., Mabry R. (2011). Died of wounds on the battlefield: Causation and implications for improving combat casualty care. J. Trauma-Inj. Infect. Crit. Care.

[60] Eastridge B.J., Mabry R.L., Seguin P., Cantrell J., Tops T., Uribe P., Mallett O., Zubko T., Oetjen-Gerdes L., Rasmussen T.E. (2012). Death on the battlefield (2001–2011): Implications for the future of combat casualty care. J. Trauma Acute Care Surg..

[61] Biggs T.C. (2011). (<C>ABC): How the British Military deals with trauma. J. Trauma-Inj. Infect. Crit. Care.

[62] Hodgetts T.J., Mahoney P.F., Russell M.Q., Byers M. (2006). ABC to <C>ABC: Redefining the military trauma paradigm. Emerg. Med. J..

[63] Butler F.K. (2017). Two decades of saving lives on the battlefield: Tactical combat casualty care turns 20. Mil. Med..

[64] Ferrada P., Dissanaike S. (2023). Circulation First for the Rapidly Bleeding Trauma Patient—It Is Time to Reconsider the ABCs of Trauma Care. JAMA Surg..

[65] Kragh J.F., Littrel M.L., Jones J.A., Walters T.J., Baer D.G., Wade C.E., Holcomb J.B. (2011). Battle casualty survival with emergency tourniquet use to stop limb bleeding. J. Emerg. Med..

[66] Welch M., Barratt J., Peters A., Wright C. (2020). Systematic review of prehospital haemostatic dressings. BMJ Mil. Health.

[67] Wedmore I., McManus J.G., Pusateri A.E., Holcomb J.B. (2006). A special report on the chitosan-based hemostatic dressing: Experience in current combat operations. J. Trauma-Inj. Infect. Crit. Care.

[68] Hatamabadi H.R., Zarchi F.A., Kariman H., Dolatabadi A.A., Tabatabaey A., Amini A. (2015). Celox-coated gauze for the treatment of civilian penetrating trauma: A randomized clinical trial. Trauma Mon..

[69] Brown M.A., Daya M.R., Worley J.A. (2009). Experience with Chitosan Dressings in a Civilian EMS System. J. Emerg. Med..

[70] Sigal A., Martin A., Ong A. (2017). Availability and use of hemostatic agents in prehospital trauma patients in Pennsylvania translation from the military to the civilian setting. Open Access Emerg. Med..

[71] Brohi K., Singh J., Heron M., Coats T. (2003). Acute Traumatic Coagulopathy. J. Trauma.

[72] Hess J.R., Brohi K., Dutton R.P., Hauser C.J., Holcomb J.B., Kluger Y., Mackway-Jones K., Parr M.J., Rizoli S.B., Yukioka T. (2008). The coagulopathy of trauma: A review of mechanisms. J. Trauma-Inj. Infect. Crit. Care.

[73] Davenport R.A., Brohi K. (2016). Cause of trauma-induced coagulopathy. Curr. Opin. Anaesthesiol..

[74] Brill J.B., Brenner M., Duchesne J., Roberts D., Ferrada P., Horer T., Kauvar D., Khan M., Kirkpatrick A., Ordonez C. (2021). The Role of TEG and ROTEM in Damage Control Resuscitation. Shock.

[75] Morrison J.J., Dubose J.J., Rasmussen T.E., Midwinter M.J. (2012). Military application of tranexamic acid in trauma emergency resuscitation (MATTERs) study. Arch. Surg..

[76] Roberts I., Shakur H., Coats T., Hunt B., Balogun E., Barnetson L., Cook L., Kawahara T., Perel P., Prieto-Merino D. (2013). The CRASH-2 trial: A randomised controlled trial and economic evaluation of the effects of tranexamic acid on death, vascular occlusive events and transfusion requirement in bleeding trauma patients. Health Technol. Assess..

[77] Devereaux P., Marcucci M., Painter T.W., Conen D., Lomivorotov V., Sessler D.I., Chan M.T., Borges F.K., Martínez-Zapata M.J., Wang C.Y. (2022). Tranexamic Acid in Patients Undergoing Noncardiac Surgery. N. Engl. J. Med..

[78] Myles P.S., Smith J.A., Forbes A., Silbert B., Jayarajah M., Painter T., Cooper D.J., Marasco S., McNeil J., Bussières J.S. (2017). Tranexamic Acid in Patients Undergoing Coronary-Artery Surgery. N. Engl. J. Med..

[79] Zhao H., Liu S., Wu Z., Zhao H., Ma C. (2019). Comprehensive assessment of tranexamic acid during orthognathic surgery: A systematic review and meta-analysis of randomized, controlled trials. J. Cranio-Maxillofac. Surg..

[80] Lewis S.R., Pritchard M.W., Evans D.J., Butler A.R., Alderson P., Smith A.F., Roberts I. (2018). Colloids versus crystalloids for fluid resuscitation in critically ill people. Cochrane Database Syst. Rev..

[81] Cantle P.M., Cotton B.A. (2017). Balanced Resuscitation in Trauma Management. Surg. Clin. N. Am..

[82] Spinella P.C., Perkins J.G., Grathwohl K.W., Beekley A.C., Holcomb J.B. (2009). Warm Fresh Whole Blood Is Independently Associated with Improved Survival for Patients with Combat-Related Traumatic Injuries. J. Trauma.

[83] Torres C.M., Kent A., Scantling D., Joseph B., Haut E.R., Sakran J.V. (2023). Association of Whole Blood with Survival among Patients Presenting with Severe Hemorrhage in US and Canadian Adult Civilian Trauma Centers. JAMA Surg..

[84] Futran N.D., Farwell D.G., Smith R.B., Johnson P.E., Funk G.F. (2005). Definitive management of severe facial trauma utilizing free tissue transfer. Otolaryngol.-Head. Neck Surg..

[85] Brennan J., Gibbons M.D., Lopez M., Hayes D., Faulkner J., Eller R.L., Barton C. (2011). Traumatic airway management in operation Iraqi freedom. Otolaryngol.-Head. Neck Surg..

[86] Keller M.W., Han P.P., Galarneau M.R., Brigger M.T. (2015). Airway Management in Severe Combat Maxillofacial Trauma. Otolaryngol.-Head. Neck Surg..

[87] Hayreh S.S., Kolder H.E., Weingeist T.A. (1980). Central Retinal Artery Occlusion and Retinal Tolerance Time. Ophthalmology.

[88] Kelly E.W., Fitch M.T. (2013). Recurrent spontaneous globe subluxation: A case report and review of manual reduction techniques. J. Emerg. Med..

[89] Tse D.T. (2000). A simple maneuver to reposit a subluxed globe. Arch. Ophthalmol..

[90] Blanch R.J., Bishop J., Javidi H., Murray P.I. (2019). Effect of time to primary repair on final visual outcome after open globe injury. Br. J. Ophthalmol..

[91] Blanch R.J., McMaster D., Patterson T.J. (2024). Management of open globe injury: A narrative review. Eye.

[92] Dhillon A., Ahmad M.S.Z., Breeze J., Blanch R.J. (2020). Prolonged deployed hospital care in the management of military eye injuries. Eye.

[93] Powers D.B., Robertson O.B. (2005). Ten common myths of ballistic injuries. Oral Maxillofac. Surg. Clin. N. Am..

[94] Will M.J., Goksel T., Stone C.G., Doherty M.J. (2005). Oral and maxillofacial injuries experienced in support of operation Iraqi Freedom I and II. Oral Maxillofac. Surg. Clin. N. Am..

[95] Keblish D.J., DeMaio M. (1998). Early pulsatile lavage for the decontamination of combat wounds: Historical review and point proposal. Mil. Med..

[96] Knappe K., Lunz A., Bülhoff M., Schonhoff M., Renkawitz T., Kretzer J.P., Jaeger S. (2022). Pulsatile lavage systems and their potential to penetrate soft tissue. Eur. J. Trauma Emerg. Surg..

[97] Bath M.F., Suresh R., Davies J., Machesney M.R. (2021). Does pulsed lavage reduce the risk of surgical site infection? A systematic review and meta-analysis. J. Hosp. Infect..

[98] Nikfarjam M., Kimchi E.T., Gusani N.J., Avella D.M., Shereef S., Staveley-O’Carroll K.F. (2009). Reduction of surgical site infections by use of pulsatile lavage irrigation after prolonged intra-abdominal surgical procedures. Am. J. Surg..

[99] Powers D.B., Will M.J., Bourgeois S.L., Hatt H.D. (2005). Maxillofacial trauma treatment protocol. Oral Maxillofac. Surg. Clin. N. Am..

[100] Reed B.E., Hale R.G. (2010). Training Australian military health care personnel in the primary care of maxillofacial wounds from improvised explosive devices. BMJ Mil. Health.

[101] Scott P.T., Petersen K., Fishbain J., Craft D.W. (2004). *Acinetobacter* *baumannii*. JAMA-J. Am. Med. Assoc..

[102] Higgins P.G., Hagen R.M., Podbielski A., Frickmann H., Warnke P. (2020). Molecular Epidemiology of Carbapenem-Resistant *Acinetobacter baumannii* Isolated from War-Injured Patients from the Eastern Ukraine. Antibiotics.

[103] Robertson B., Manson P.N. (1998). The importance of serial debridement and “second-look” procedures in high-energy ballistic and avulsive facial injuries. Oper. Tech. Plast. Reconstr. Surg..

[104] Smith T., Evans J., Moriel K., Tihista M., Bacak C., Dunn J., Rajani R., Childs B. (2022). Cost of OR Time is $46.04 per Minute. J. Orthop. Bus..

[105] Knackstedt R., Oliver J., Gatherwright J. (2020). Evidence-based perioperative nutrition recommendations: Optimizing results and minimizing risks. Plast. Reconstr. Surg..

[106] Turk J.B., Vuillemin T., Raveh J. (1994). Revascularized Bone Grafts for Craniofacial Reconstruction. Otolaryngol. Clin. N. Am..

[107] Soto E., Strong E.B. (2021). Reconstructive management of gunshot wounds involving the face. Craniomaxillofac. Trauma Reconstr..

[108] Gurunluoglu R., Glasgow M., Williams S.A., Gurunluoglu A., Antrobus J., Eusterman V. (2012). Functional reconstruction of total lower lip defects using innervated gracilis flap in the setting of high-energy ballistic injury to the lower face: Preliminary report. J. Plast. Reconstr. Aesthetic Surg..

[109] Salman S.O., Fernandes R.P., Rawal S.R. (2017). Immediate Reconstruction and Dental Rehabilitation of Segmental Mandibular Defects: Description of a Novel Technique. J. Oral Maxillofac. Surg..

[110] Weyh A.M., Fernandes R.P. (2021). Narrative review: Fibula free flap, indications, tips, and pitfalls. Front. Oral Maxillofac. Med..

[111] Fernandes R.P., Quimby A., Salman S.O. (2017). Comprehensive Reconstruction of Mandibular Defects with Free Fibula Flaps and Endosseous Implants. J. Diagn. Treat. Oral Maxillofac. Pathol..

[112] Thorne C.H. (1992). Gunshot Wounds to the Face. Clin. Plast. Surg..

[113] Grass J.S., Antonyshyn O., Phillips J.H. (1991). Early definitive bone and soft-tissue reconstruction of major gunshot wounds of the face. Plast. Reconstr. Surg..

[114] Devauchelle B., Badet L., Lengelé B., Morelon E., Testelin S., Michallet M., D’Hauthuille C., Dubernard J.-M. (2006). First human face allograft: Early report. Lancet.

[115] Homsy P., Huelsboemer L., Barret J.P., Blondeel P., Borsuk D.E., Bula D., Gelb B., Infante-Cossio P., Lantieri L., Mardini S. (2024). An Update on the Survival of the First 50 Face Transplants Worldwide. JAMA Surg..

[116] Longo B., Pomahac B., Giacalone M., Cardillo M., Cervelli V. (2023). 18 years of face transplantation: Adverse outcomes and challenges. J. Plast. Reconstr. Aesthetic Surg..

[117] Longo B., Alberti F.B., Pomahac B., Pribaz J.J., Meningaud J.-P., Lengelé B., Özkan Ö., Özkan Ö., Barret J.P., Lassus P. (2024). International consensus recommendations on face transplantation: A 2-step Delphi study. Am. J. Transplant..

[118] Ducic Y., Oxford L., Smith J. (2019). Management of facial nerve injuries after ballistic trauma. Facial Plast. Surg..

[119] Ziccardi V.B., Steinberg M.J. (2007). Timing of microsurgical repair of trigeminal nerve injuries. J. Oral Maxillofac. Surg..

[120] Steinsapir K.D., Goldberg R.A. (2011). Traumatic optic neuropathy: An evolving understanding. Am. J. Ophthalmol..

[121] McMillan D.C., McMillan K.B., Viozzi C.F., Shivers P., Gellrich N.C., Powers D.B. (2025). A New Hope: Patient-Specific Bone-Anchored Subperiosteal Implants: Perspective of 3 US Institutions on This Resurrected Treatment Modality. J. Oral Maxillofac. Surg..

[122] Gellrich N.C., Korn P., Neuhaus M., Lentge F., Jehn P., Rahlf B. (2025). Long-Term Survival of Subperiosteal Implants: Meta-Analysis and Current Status of Subperiosteal Implants for Dental Rehabilitation. Oral Maxillofac. Surg. Clin. N. Am..

[123] Menick F.J., Salibian A. (2011). Microvascular repair of heminasal, subtotal, and total nasal defects with a folded radial forearm flap and a full-thickness forehead flap. Plast. Reconstr. Surg..

[124] Lannon D.A., Novak C.B., Neligan P.C. (2009). Resurfacing of colour-mismatched free flaps on the face with split-thickness skin grafts from the scalp. J. Plast. Reconstr. Aesthetic Surg..

[125] Clark N., Birely B., Manson P.N., Slezak S., Kolk C.V., Robertson B., Crawley W. (1996). High-energy ballistic and avulsive facial injuries: Classification, patterns, and an algorithm for primary reconstruction. Plast. Reconstr. Surg..

[126] Batstone M.D., Fox C.M., Dingley M.E., Cornelius C.P. (2013). Cosmetic Tattooing of Free Flaps following Head and Neck Reconstruction. Craniomaxillofac. Trauma Reconstr..

[127] Castro-Núñez J., Van Sickels J.E. (2017). Secondary reconstruction of maxillofacial trauma. Curr. Opin. Otolaryngol. Head. Neck Surg..

[128] Coleman S.R., Katzel E.B. (2015). Fat Grafting for Facial Filling and Regeneration. Clin. Plast. Surg..

[129] Arcuri F., Brucoli M., Baragiotta N., Stellin L., Giarda M., Benech A. (2013). The Role of Fat Grafting in the Treatment of Posttraumatic Maxillofacial Deformities. Craniomaxillofac. Trauma Reconstr..

[130] Kim S.W. (2021). Management of keloid scars: Noninvasive and invasive treatments. Arch. Plast. Surg..

[131] Monstrey S., Middelkoop E., Vranckx J.J., Bassetto F., Ziegler U.E., Meaume S., Téot L. (2014). Updated Scar Management Practical Guidelines: Non-invasive and invasive measures. J. Plast. Reconstr. Aesthetic Surg..

[132] Fowler K.A., Dahlberg L.L., Haileyesus T., Annest J.L. (2015). Firearm injuries in the United States. Prev. Med..

[133] Bisson J.I., Shepherd J.P., Dhutia M. (1997). Psychological sequelae of facial trauma. J. Trauma-Inj. Infect. Crit. Care.

[134] Lal B., Ganesh K., Alagarsamy R., Gupta S., Kumar M., Barathi A. (2024). Post-traumatic stress disorder in maxillofacial trauma victims- A systematic review and meta-analysis. J. Stomatol. Oral Maxillofac. Surg..

[135] Walshaw E.G., Taylor R., Anderson J., Sexton P., Parmar J.D., Carter L.M. (2022). The psychological sequelae of maxillofacial trauma: A scoping review of the literature. Br. J. Oral Maxillofac. Surg..

[136] Sahni V. (2018). Psychological Impact of Facial Trauma. Craniomaxillofac. Trauma Reconstr..

[137] Tsur N., Talmy T., Radomislensky I., Almog O., Gendler S. (2023). Traumatic maxillofacial injuries: Patterns, outcomes, and long-term follow-up of a military cohort. Dent. Traumatol..

[138] Phillips C.J., LeardMann C.A., Gumbs G.R., Smith B. (2010). Risk factors for posttraumatic stress disorder among deployed US male marines. BMC Psychiatry.

[139] Brewin C.R., Andrews B., Valentine J.D. (2000). Meta-analysis of risk factors for posttraumatic stress disorder in trauma-exposed adults. J. Consult. Clin. Psychol..

[140] Kish V., Lansdown R. (2000). Meeting the Psychosocial Impact of Facial Disfigurement: Developing a Clinical Service for Children and Families. Clin. Child Psychol. Psychiatry.

[141] Rahtz E., Bhui K., Hutchison I., Korszun A. (2018). Are facial injuries really different? An observational cohort study comparing appearance concern and psychological distress in facial trauma and non-facial trauma patients. J. Plast. Reconstr. Aesthetic Surg..

[142] Rankin M., Borah G.L. (2003). Perceived functional impact of abnormal facial appearance. Plast. Reconstr. Surg..

[143] Bennett M.H., Trytko B., Jonker B. (2012). Hyperbaric oxygen therapy for the adjunctive treatment of traumatic brain injury. Cochrane Database Syst. Rev..

[144] Sakovich Y.e.F., Iskra Y.u.V., Maltseva L.A. (2015). Hyperbaric Oxygenation in the Complex of Intensive Care for Gunshot Wounds and Blast Injuries. Emerg. Med..

[145] Jay Chapman A., McClain J. (1984). Wandering missiles: Autopsy study. J. Trauma.

[146] Baum G.R., Baum J.T., Hayward D., MacKay B.J. (2022). Gunshot Wounds: Ballistics, Pathology, and Treatment Recommendations, with a Focus on Retained Bullets. Orthop. Res. Rev..

[147] Weiss D., Tomasallo C.D., Meiman J.G., Alarcon W., Graber N.M., Bisgard K.M., Anderson H.A. (2017). Elevated Blood Lead Levels Associated with Retained Bullet Fragments—United States, 2003–2012. MMWR Morb. Mortal. Wkly. Rep..

[148] Nguyen A., Schaider J.J., Manzanares M., Hanaki R., Rydman R.J., Bokhari F. (2005). Elevation of blood lead levels in emergency department patients with extra-articular retained missiles. J. Trauma-Inj. Infect. Crit. Care.

[149] Kosnett M.J., Wedeen R.P., Rothenberg S.J., Hipkins K.L., Materna B.L., Schwartz B.S., Hu H., Woolf A. (2007). Recommendations for medical management of adult lead exposure. Environ. Health Perspect..

[150] Apte A., Bradford K., Dente C., Smith R.N. (2019). Lead toxicity from retained bullet fragments: A systematic review and meta-analysis. J. Trauma Acute Care Surg..

[151] Eward W.C., Darcey D., Dodd L.G., Zura R.D. (2011). Case report: Lead toxicity associated with an extra-articular retained missile 14 years after injury. J. Surg. Orthop. Adv..

[152] Peled M., Leiser Y., Emodi O., Krausz A. (2012). Treatment protocol for high velocity/high energy gunshot injuries to the face. Craniomaxillofac. Trauma Reconstr..

[153] Kihtir T., Ivatury R.R., Simon R.J., Nassoura Z., Leban S. (1993). Early management of civilian gunshot wounds to the face. J. Trauma-Inj. Infect. Crit. Care.

[154] Suominen E., Tukiainen E. (2001). Close-range shotgun and rifle injuries to the face. Clin. Plast. Surg..

[155] Shvyrkov M.B. (2013). Facial gunshot wound debridement: Debridement of facial soft tissue gunshot wounds. J. Cranio-Maxillofac. Surg..

[156] (2026). US Military Joint Trauma System Clinical Practice Guidelines. https://jts.health.mil/index.cfm/CPGs/cpgs.

[157] Breeze J., Bowley D.M., Combes J.G., Baden J., Rickard R.F., DuBose J., Powers D.B. (2019). Facial injury management undertaken at US and UK medical treatment facilities during the Iraq and Afghanistan conflicts: A retrospective cohort study. BMJ Open.

[158] Walshe T.M. (2016). On Wounds of the Head: Hippocrates. Neurol. Concepts Anc. Greek Med..

[159] Strong E.B., Tollefson T.T. (2013). Intraoperative Use of CT Imaging. Otolaryngol. Clin. N. Am..

[160] Klug C., Schicho K., Ploder O., Yerit K., Watzinger F., Ewers R., Baumann A., Wagner A. (2006). Point-to-point computer-assisted navigation for precise transfer of planned zygoma osteotomies from the stereolithographic model into reality. J. Oral Maxillofac. Surg..

[161] Schreurs R., Klop C., Gooris P.J.J., Maal T.J.J., Becking A.G., Dubois L. (2022). Critical appraisal of patient-specific implants for secondary post-traumatic orbital reconstruction. Int. J. Oral Maxillofac. Surg..

[162] Roser S.M., Ramachandra S., Blair H., Grist W., Carlson G.W., Christensen A.M., Weimer K.A., Steed M.B. (2010). The accuracy of virtual surgical planning in free fibula mandibular reconstruction: Comparison of planned and final results. J. Oral Maxillofac. Surg..

[163] CRASH-2 Trial Collaborators (2010). Effects of tranexamic acid on death, vascular occlusive events, and blood transfusion in trauma patients with significant haemorrhage (CRASH-2): A randomised, placebo-controlled trial. Lancet.

