# Supplementary material for: Management Protocol for Ballistic and Other High-Energy Avulsive Facial Injuries—An Update for the 21st Century
Source: Craniomaxillofac Trauma Reconstr. 2026 Mar 3;19(1):14. doi: 10.3390/cmtr19010014 (PMC13026054; doi:10.3390/cmtr19010014)
Supplement: Supplementary file 1 [file cmtr-19-00014-s001.zip › cmtr-3981286-supplementary.pdf]

Table S1. Summary of included literature reviewed for the fabrication of this manuscript.

First Author	Country of Study	Year	Study Design	Number of Patients	Mean Age	Setting of Injury	Type of Firearm	Timing of Intervention	Follow-Up Period	Major Findings	Aesthetic Outcomes	Complications
Shvyrkov	Russia	2013	Case Series	502 soft tissue injuries	Not reported	Afghanistan – Russia War 1981-1985	Not specified; bullets, blast, and shrapnel	Day of injury – Day 15	Not reported	Recommended radical primary surgical debridement	NA	
Uçak	Syria	2020	Cross-sectional retrospective clinical cohort	Patients with fasciocutaneous transposition flap (cervical region) = 108; patients with interpolation flap (frontotemporal region) = 44; patients with forehead rotation flap = 34	38.1 +/- 27.9 years (ranged 1.5–78 years)	Syrian War	Not reported	Days later due to being away from battlefields	Not reported	Injured Facial Regions: Maxilla-Zygoma = 71, Orbital-Nose = 59, and Mandibula-Teeth =56 fasciocutaneous transposition flap, interpolation flap, and forehead rotation flap are rare flaps but gave great outcomes. No signs of necrosis in war surgery	Surgeons considered best functional and aesthetic outcome when choosing flap type	
Peleg et al	USA	2010	Retrospective Cohort	N = 92 sustaining GSWs to the mandible b/t 1999-2009; of this, N = 30 comminuted and displaced and N= 62 with minimal or no displacement; of the 30 with comminuted, they underwent similar management (dental impression w/ study models and model surgery-->IMF with Erich arch bars to stabilize--> OR for aggressive debridement and reconstruction using rigid titanium reconstruction plates + soft tissue reconstruction using adjacent tissue transfer or myocutaneous flaps--> IMF removal	only reported for N= 92 cohort --> mean = 36 with range from 13-72	assault in US/ self-inflicted ("close-range")	Handguns caused most injuries (72), with assault rifles (9) and shotguns (11)	7 days; at some undisclosed point all patients with continuity defects underwent mandibular bone reconstruction with corticocancellous bone grafts, vestibuloplasty, and dental implant rehabilitation	NA	Only reported methods for N= 30 with comminuted, they underwent similar management (dental impression w/ study models and model surgery-->IMF with Erich arch bars to stabilize--> OR for aggressive debridement and IMMEDIATE reconstruction--> continuity defects underwent reconstruction using rigid titanium reconstruction plates + soft tissue reconstruction using adjacent tissue transfer or myocutaneous flaps --> IMF removal (1 total surgery)... at some undisclosed point all patients with continuity defects underwent mandibular bone reconstruction with corticocancellous bone grafts, vestibuloplasty, and dental implant rehabilitation	NA	33.3% complication rate and there were 3 deaths
Guerrier et al	Iraq	2012	Retrospective Cohort	Patients with iliac crest bone graft reconstructions = 35	33 + 19	Combat in Middle East	not specified; bullets, blast, and shrapnel	mean = 824 days, range = 45-3814 days	17 months	First stage: exploration of the mandibular defects, removal of fixations or miniplates, and debridement of the wound and bone. Occlusion was re-established using the remaining teeth and the wounds were closed in layers. Antibiotics were given for any cultures collected during this time. Second stage started 6 weeks after completion of antibiotics and included a new exploration and reconstruction of the defect using corticocancellous bony chips from the hip that was fixed to the reconstruction plate with bicortical screws. The final stage was 4 months after re-exploration and involved removal of the reconstruction plate.<br><br>The quality and quantity of soft tissues surrounding the graft were indicators of success of the procedure. Scars and poor quality soft tissue led to compromised blood supply and higher risk of reconstruction failure. Neither the anatomical site of the defect, nor sex, diabetes, smoking, time from injury to operation, mechanism of injury, size of defect, type of fixation, or number of operations significantly increased the risk of	failure.<br><br>The mean number of reconstructive surgeries was 3 (range 1-8).	Not discussed

								Extraoral and intraoral exposure in	one and fractur	e. Five patients developed seromas at the donor site.		
Wei et al	China	2013	Retrospective Cohort	Patients with complete foreign body removal = 40, patients with partial	24.1 + 12.5	Modern war in China	pistols, rifles, machine guns, shells, land mines, grenades,	5 of 54 patients received debridement within 6 h after injury, 34	not discussed	Reconstruction/treatment included direct-visual complete foreign body removal under the guide of the stereotaxis or neuro navigation technique. Patients receiving	The immediate emergency surgical approach was	Total nasal obstruction when no intranasal stenting and

				foreign body removal = 2, patients not receiving removal treatment = 1			detonators, shotguns, air guns	between 6 h and 8 h, and 15 after 8 h. No revision surgeries discussed.		debridement within 8 h after injury got a higher recovery rate than those receiving debridement after 8 h of injury. Reasonably deciding on the operative mode and approach for intracranial residing foreign body removal, and increasing vigilance regarding concomitant injuries were also necessary for improvement of overall treatment.	found to protect the lacerated tissues, stop the bleeding, reduce the subsequent disfigurement	scaffolding of lacertid tissue was used. A delay in that action for more than 24 h also appeared to be associated with increased tissue necrosis.
Fernandez et al	USA	2007	Retrospective Chart review	n=4 treated with lateral circumflex femoral artery perforator flap	20, 32, 43, 40 (cases described independently)	self-inflicted (3), assault (1)	NA	initial surgery was either emergent or timing not specified; revision surgeries did not specify timing	NA	Using Lateral Circumflex Femoral Artery Perforator (LCFAP) flaps for reconstruction of defects secondary to GSWs has 100% success rate with no partial necrosis of flaps, no donor site complications, and successful primary closure; case 1 had 2 surgeries to debulk flap in addition to "several" other reconstructive surgeries	restoration of facial profile and contour	case 1: uneventful recovery; case 2: uneventful recovery; case 3 had extreme agitation due to narcotic withdrawal and had total flap loss during initial surgery using osteocutaneous fibula flap, but no reported complications after LCFAP; case 4 initially treated with cervicofacial flap that did not produce desirable outcomes, but no reported complications since LCFAP placement
Kummoona et al	Iraq	2006	Cross-Sectional	N = 100 total; N=13 soft tissue injury, N = 87 skeletal injury	mean = 37.5 (range 3-72, no SD)	combat in Middle East	rifle bullets (49%) followed by fragments (29%), handgun bullets (15%), airgun pellets (6%), and shotgun (1%).	NA "primary surgery was executed as early as possible"	Not specified (some photos are 3-8 months post-op)	closed reduction with indirect fixation and open reduction with indirect fixations; outcomes not discussed; surgery count not discussed	NA	3% mortality due to complications related to associated severe head injuries
Eser et al	Turkey	2016	Cross-Sectional	N = 7 who underwent reconstruction with free osteoseptocutaneous fibula flap (FOCF) for mandibular defect and pre or nonexpanded scalp flap for beardless facial skin	29.1 (range 15-45 no SD)	NA	NA	immediate surgery for debridement, reconstruction plates for mandibular defects, and wound closure (discharged after medical stabilization)--> 6 months later composite reconstruction began with FOCF --> 3 months later scalp flap pre-expansion for N=3 (2 stages) --> 2.5 months later beard reconstruction --> 1 month later continued beard reconstruction --> "last" operation (no timing) finalized beard reconstruction (timeline may vary for N=4 patients who declined pre-expansion but it is not specified in article)	2-5 years, average 3.5 years	All patients underwent reconstruction with a free osteoseptocutaneous fibula flap (FOCF) for the composite mandibular defect and a pre or nonexpanded temporal artery-based scalp flap for beardless facial skin. ~7 reported surgeries/patient who underwent pre-expansion (N=3) and ~5 for those who did not (N=4) (differences between #surgeries bt patients not well described, particularly for # of operations for N=3 patients who underwent pre-expansion)	restoration of mandibular contour was deemed satisfactory (N=4), acceptable (N=2), and unsatisfactory (N=1)	N = 2 post-op venous thrombosis (flap survived); late expander exposition was observed in 2 pre-expanded scalp flaps
Firat and Geyik	Turkey	2013	Case Series	patients with free radial forearm osteocutaneous flap + rib grafts = 1, free fibular osteocutaneous flap = 2, rib bone grafts =	26 + 7; patients with free radial forearm osteo-cutaneous flap + rib grafts = 17, free fibular osteocutaneou	self-inflicted, accidental, assaults	shotgun, pistol	The reconstruction procedure using the free flaps was performed within 2 months on average for all other patients.	Mean follow-up period was 4.2 years	All of the patients received a tracheostomy. Reconstruction was performed in the second surgery for 10 patients. Necrotic bone, soft tissues, and foreign bodies were debrided during the first surgical intervention. Soft tissue defects were reconstructed using	All patients were closely monitored for scar development. Consecutive fat injections,	Nasal vestibular stenosis, nasal collapse, saddle nose, scars, mandibular plate and bony exposure, left eye

				2, iliac bone graft = 3, free radial forearm osteocutaneous flap = 3, none = 1	s flap = 25 + 2, rib bone grafts = 25 + 4, free radial forearm osteo-cutaneous flap = 28 +9, none = 33					the local flaps. For cases with large soft tissue defect, reconstruction was performed in the first intervention using the radial forearm fasciocutaneous flap. The multi-fragmented fractures on the front wall of the maxilla were kept for 9 days, on average, after placing a Foley catheter inside the maxillary sinus and inflating with saline. Bony fixation was obtained using Doyle nasal splints for the passage and external nasal casting.<br><br>The first reconstruction surgery must be performed using the shortest and most practical methods following precise debridement.	steroid injections, scar revisions, and laser treatments to the depressed scar areas were applied to all patients beginning 6 months after the operation.	socket contraction orbital abscess, retrogenia, oral incompetence, dark discoloration of the flap, trismus.
Riyadh et al	Iraq	2018	Case Series	Patients with no soft tissue or orbital rim loss (single stage reconstruction) = 7, Patients with soft tissue or orbital rim loss (debridement, irrigation, packing; then reconstruction later) = 9; groups also broken down into bullet and blast injuries	29.8 y/o (range 19–48)	Iraqi army vs Islamic State of Iraq and Syria (ISIS) conflict	not reported	mean time: injury to hospital admission - 12 hours<br>mean time: injury to the surgery - 16 hours.	Mean 3 months	titanium mesh to reconstruct orbital walls bone grafts from iliac crest<br><br>w/o tissue loss --> single stage reconstruction<br>w/ tissue loss --> debridement, irrigation, packing --> reduction/reconstruction with graft and/or flap	2 not satisfied, 10 satisfied, 4 very satisfied	death (2), Infection (1), Wound dehiscence (1), Enophthalmos (2), Flap failure (1), Nerve sensibility disturbances (5), poor Vision outcomes (13), Oronasal fistula (1)
Rana et al	Pakistan	2014	Randomized Control Trial	patients with open reduction and internal fixation = 30; patients with closed reduction and maxillomandibular fixation = 30	27.4 y/o (SD 10.7)	Civilian injuries; Not explicitly said for each case but says, "Main causes of the gunshot injuries in this part of the world are violent crimes, domestic violence, accidental discharge of bullet, suicidal attempts and air shooting"	Not mentioned	mean duration of 5.8 days (SD 3.2) (day of up to 15 days)	3 to 10 months	Mandible sites affected: 28 body (47%), 18 symphysis/parasymphysis (30%), and 14 angle (23%)<br><br>it was noted that open reduction had less complications compared to closed reduction<br>Open reduction and internal fixation allowed quicker mandibular mobility, with good functional and aesthetic results<br><br>open reduction and internal fixation (ORIF) better for mandible fractures without continuity defect from gunshots		Closed reduction and maxillomandibular fixation (CRMF) and open reduction and internal fixation (ORIF)<br><br>Infection (CRMF-0; ORIF-2), Malocclusion (CRMF-1; ORIF-3), malunion (CRMF-6; ORIF-1), facial asymmetry (CRMF-7; ORIF-1), sequestration of bone (CRMF-1; ORIF-2), exposed plate (CRMF-0; ORIF-2)
Sadda	Iraq & Iran	2003	Case Series	Patients with Debridement and primary closure and/or removal of teeth and foreign body = 108, Patients with Intermaxillary fixation = 81, Patients with Interosseous wires = 23, Patients with K-wires = 21, Patients with Circum-mandibular wires = 7, Patients with External fixation = 2, Patients with Plates = 2, Patients with Metal mesh = 1, Patients with Twisted wires = 2, Patients with Packing open = 5, Patients with Intranasal portex<br><br>tubes = 7	N/A	Iraq-Iran war	80.7% shrapnel, 19.3% bullets	Immediate (4-6 hours) but sometimes complications and delays (due to war)	N/A	Claims no deaths due to rapid evacuation, immediate resuscitation, proper organization, and postoperative care<br>More shrapnel injuries than bullet<br>Simple debridement, removal of teeth or foreign bodies, followed by primary closure (36%)<br>Eyelet wiring and intermaxillary fixation (27%)<br>intraosseous wiring (7.7%)<br>Kirschner wire to support the soft tissues and the tongue in cases of severe osseous loss of the body and/or symphysis of the mandible (7%)<br><br>The amount of soft and hard tissue injury was often 30–40 times the size of the bullet.<br>Shrapnel had less tissue damage.		

Ucak	Turkey	2019	Prospective Cohort	patients with maxillofacial injuries = 112	29.1 + 12	combat in middle east	missile and shrapnel	n/a	unable to report due to patients being in constant migration, unable to reach; limited follow up	debridement and primary closure (n=76), teeth and foreign body (n = 50), IMF (n=56), interosseous wires (n=62), external fixation (n=5), plates (n=105), titanium mesh (n= 75) bone graft iliac or fibula (n=38)	n/a	n/a
Jose et al	India	2019	Retrospective Chart Review	isolated maxillofacial injuries =16	29.6 +- 4.2	combat in middle east	assault rifle	operated within first 24-48 hours of injury	mean hospital stay = 40 days	OVERALL: one staged debridement followed by reconstruction--> osteosynthesis with standard AO plates in 15/16 patients. secondary reconstruction such as microvascular reconstruction using radial forearm flap and fibular free flap in 3 patients. reconstruction using iliac crest graft in 1 patient. ..minimized periosteal stripping during hardware application, used broad spectrum IV antibiotics, clean paraffin dressings, copious irrigation	outcome of treatment is satisfactory if early debridement and reconstruction is planned	tracheostomy to manage airway = 8 ; external carotid artery ligation = 1 ; nerve injury = 7 ; scar contracture = 9 (caused trismus in 2, microstomia in 1, restricted neck movements in 2, and incompetent lips in 3) ' salivary gland injury = 3
Orthopoulos et al	USA	2013	Retrospective Cohort	patients admitted to Level 1 academic trauma center = 155 (isolated Gunshot wound to the face = 115; urgent airway control = 90, urgent bleeding control = 41)	patients admitted = 26 + 11	assault in United States, self-inflicted	not specified	Surgeries were divided by early (n = 37) and late (n = 21) repair set at 48 hours, but no relation could be made between timing and outcomes. All eye operations were performed within 24 hours. All other patients underwent their first operation within 2 +/- 3 days after injury.<br><br>Revision surgeries included microvascular osseous reconstruction in cases of significant bone loss. The most commonly reported bone grafts were the fibula osteo-cutaneous free flap and the radial forearm free flap. The anterolateral thigh free flap was also used for soft tissue defects.	5 +/- 9 months	Simple methods, such as orotracheal intubation and packing, are typically sufficient for successful management. About half of the patients needed further surgery, with infrequent morbidity.<br><br>Although the timing of definitive facial reconstruction is controversial, they suggested that early definitive, thin, fasciocutaneous free flaps were ideal for resurfacing large wounds and mucosal deficits in areas such as the zygoma. In more severe cases, where significant osseous reconstruction is required, they stated that long, definitive procedures should be deferred to a later time when the swelling has decreased and the patient is not acutely ill.	4 patients had. substandard cosmesis (keloid formation and skin atrophy).	14 patients suffered from complications. 9 had complications of fractured facial bones (3 malunions, 6 osteomyelitis cases). 2 patients had an abscess form in the mandible. 1 patient had an oroantral fistula. 2 patients had a persistent cerebrospinal leak. 10 patients developed eye-specific problems. 6 patients had general complications (pneumonia).
Siddiqui et al	Pakistan	2020	Randomized Control Trial	N=40 with comminuted mandible fracture, divided into: ORIF with AO mini-plates treatment = 20 ; MMF with eric arch bars treatment = 20	36.35 +- 12.9	NA	NA	NA	8 weeks	ORIF is superior to MMF in managing comminuted mandibular fractures; N=19 showed callus formation, 14 of which received ORIF and 5 received MMF--> The final healing considered by 8th week was in 16 (80%) of ORIF group, and 8 (40%) MMF group	NA	delayed/non-union = 21 total (6 ORIF, 15 MMF); presence of pain and mobility = 16 total (4 ORIF, 12 MMF)
Ahmed et al	Pakistan	2018	Case Series	patients who underwent maxillofacial reconstruction with non-vascularized bone grafts = 30 (iliac crest cortico-cancellous bone graft = 15, rib graft = 11, costochondral graft = 4)	33.57 + 14.74	combat in Middle East, not all patients suffered from gunshot wounds	gunshot, blast injury	For gunshot wounds, patients underwent primary and revision surgery, with the second surgery occurring during next four to eight weeks, after residual mandibular segments are stabilized by maxillomandibular fixation to preserve	6 months	Reconstructions of maxillofacial osseous defects were achieved by different non-vascularized bone grafts, i.e. autogenous rib, costochondral bone, or corticocancellous iliac crest bone graft. Out of the 13 gunshot wounds, 9 were reconstructed with iliac crest cortico-cancellous bone, while 4 were done with rib bone graft. Over 85% of non-vascularized autogenous bone cases were considered successful.<br><br>Gunshot wound defect reconstruction was carried out in two stages. In the first stage,	All patients had adequate mouth opening (i.e. >30 mm), when evaluated 6 months after the reconstruction.	Surgical site infection and graft loosening/failure in only one case done with iliac crest bone. No donor site morbidity was observed in any patient.

								occlusion, and to prevent unnecessary pull of muscles that might result in displacement of fracture segments.<br><br>3 patients had revision surgery due to dissatisfaction with dental prosthesis (inadequate retention and stability), but timing was not specified.		site is made free of infection by removal of foreign body, devitalization of teeth and necessary debridement is carried out followed by reconstruction in second phase. Penetrating, perforating and avulsive gunshot wounds were found infected in most of the cases. Then Finally, in second stage, definitive reconstruction with non-vascularized bone grafts is performed once complete eradication of infection isensured.		
Newlands et al	USA	2003	Retrospective Chart Review	N= 90 patients with mandibular injuries due to gunshot with N=68 undergoing surgical procedure (see details below)	mean = 30, range = 7-68	assault in USA = 62, self inflicted = 21, accidental = 7	handguns (n=57), rifles (n=11), shotguns (n=14)	NA	mean follow up for 49/68 patients = 465 days	In 40 patients, MMF was used to immobilize the fractures (Thirty had arch bars alone, 5 had arch bars and interosseous wiring, and 5 were secured by 4-point fixation). 18 underwent ORIF with rigid plates (3 of these, MMF alone was used to temporarily stabilize the fracture and ORIF with rigid plates was accomplished several days later MMF and ORIF were combined in 13 of the 18 patients). 2 patients, treated initially with MMF, had reconstruction plates placed secondarily (Both of these patients had defects that required cancellous bone grafting). 10 patients, external fixation was primary (5 of these were also placed in MMF, and in 1 of these, a rigid plate was also used…In 3 of these patients, a microvascular free tissue transfer (2 fibular and 1 radial forearm osseo-cutaneous flap) was performed to replace bone	NA	Complication rates are higher when there is a mandibular continuity defect
Bukhari et al	Pakistan	2020	Case Series	gunshot wounds to face = 38--> ORIF = 17/38 (plating and trans-osseous wiring), conservative treatment = 21/38, reconstruction = 3/38 (see below)	28+- 4.98	combat in middle east	NA	NA	NA	N=3/38 underwent reconstruction --> N=1 with delayed reconstruction using rib on-lay grafts, N=2 with early reconstruction using fibular free grafts	NA	N= 15/38 with complications including: trismus (10), infection/sinusitis (10), facial nerve palsy (2), vision loss (1)
Liu et al	USA	2018	Retrospective Chart Review	N = 190 with facial fractures from GSW --> 89/190 managed in OR (details below)	mean = 29.9 (range 14-81)	Self-inflicted = 16	low velocity = 97%; intermediate velocity = 3%	NA	NA	Of the patients treated, 139 (77.7%) were able to be managed conservatively with maxillomandibular fixation whereas 32 (17.9%) required internal fixation and 8 (4.5%) required external fixation. All other patients underwent some form of bedside irrigation with conservative debridement. 9 patients required local or regional flaps for soft-tissue coverage. In 1 patient, enucleation and orbitotomy were required to remove the bullet casing and a tarsorrhaphy and a sliding lower lid flap were utilized to close the wound	NA	NA
Hoppe et al	USA	2014	Retrospective Chart Review	N=14 pediatric patients with facial fractures due to GSW, see breakdown below	16.5 (range 14-18)	assault in USA	handgun	NA	hospital stay mean = 8.2 days	7 patients ultimately went to the operating room for fracture management. The fracture treatment modalities employed were conservative management with closed techniques like MMF (n = 9), rigid internal fixation (n = 4), and the use of an external fixator device (n = 1). Minimal soft-tissue debridement was performed in 10 of the 14 patients; 2 of which presented between 6 months and 10 years post-injury with soft-tissue complications related to retained material. 4 patients underwent extensive debridement and fixation of fractures.	Defects were mainly related to loss of bone. In patient #8, there was approximately a 2-cm loss of zygoma, which necessitated replacement of debrided portions of bone as a graft	N = 3 developed abscesses around retained portions of bullet that were left in the soft tissue. N = 1 facial nerve palsy

Soto et al	USA	2021	retrospective chart review	N=28 with frontal sinus fracture injury --> N=21 managed with a vascularized filling material (7 temporal, 9	27.57	"firearm violence" in USA , did not differentiate between self-inflicted vs not	NA	4.44 ± 6.74 for N= 28 total	total hospital stay for N=28 total --> 49.71+-42.18	Free flap reconstruction is a equally efficacious alternative to traditional temporal and pericranial flaps in frontal sinus reconstruction in situations with large soft tissue loss and increased dead space. Significant difference in reconstructive	NA	most common acute major complication was CSF leak (39%), followed by sinusitis (18.5%).

				pericranial, or 5 free flap) ; N=7 managed using non-vascularized material ("other" = either fat, alloplastic or unclearly defined)						material usage when the injury included the presence of naso-frontal sinus outflow tract involvement (NFOT) displacement, with 100% of temporal graft recipients and 80% of free flap recipients presenting with NFOT displacement. Most patients who underwent filling with either pericranial (66%) material or other (86%) did not present with NFOT displacement. When looking at reconstructive strategies, the most common method was cranialization (52%, n=14), followed by obliteration (30%; n= 8), ORIF (15%, n=4) and purely endoscopic procedures (3%, n=1).<br>There was a difference in the type of filling material used for each reconstructive strategy, with pericranial flaps primarily used in cranialization (76%), temporal grafts were more likely to be used in obliteration (57%), free flaps more likely to be used in cranialization (60%) and other non-vascularized material being about as likely to be used for all three types of procedures.		The most common chronic major complication was abscess (23.5%). The most common minor complication was minor infection (14%), without associated morbidity in the 6 months following the reconstruction operation...NO flap failures
Tabakan et al	Turkey	2021	Case Series	total patients overall = 61 (patients with head/neck injuries = 24)	25.2	Combat in Middle East	high-energy firearm	Not specified in terms of timing.	Hospitalized for 18-129 days	Free flaps (ALT, radial forearm, rectus abdominis, latissimus dorsi), muscle pedicle flaps (trapezius and pectoralis major myocutaneous flaps) and fasciocutaneous perforator flaps were performed for firearm-caused soft tissue defects of the neck region. Pedicle flaps were preferred when free flaps were not feasible. Pectoral muscle, deltopectoral, and transverse cervical artery perforator flap were used in urgent cases.<br>Range of 1-4 surgeries per patient. Only 2 patients had 4 surgeries, 5 had 2 surgeries, and the rest only had 1.	Not specified, but free flaps were noted to have the best aesthetic appearance and great functional advantage.	Two patients experienced flap loss, though no other major complications; one had been treated with a free gracilis flap, the other with a free anterolateral thigh (ALT) flap. Other complications included bleeding, secondary infection, flap detachment, hematoma and seroma.
Gröbe et al	Germany	2009	Retrospective Case Control	patients with image-guided surgical removal of projectiles = 32, patients with surgical removal of projectiles without navigation assistance = 18	50 (17-77)	violent conflict, negligent handling, professional reason, suicide	shotguns, handguns, projectiles	Time between injury and first surgery not specified, although operating time as a whole was found to have significant correlation with complications.<br>No revision surgeries were noted.	at least 6 days (mean = 14.3 + 7.9)	Projectiles were removed with minimally invasive surgical access using an intraoperative imaging system (a mobile dental X-ray device for the acquisition of conventional radiographs in two planes, with reference markers attached to the surgical site). Microvascular free tissue transfer and rotational flaps for primary closure of large defects were used for soft tissue repair.	Immediate reconstruction of defects were equally effective in terms of appearance and function as routine reconstruction procedures.	The overall complication rate was 34%. There was a significant correlation between the navigated surgery vs. not-navigated surgery and the complication rate, including major bleeding, soft tissue infections, and nerve damage. There was also a correlation between operation time and postoperative complications such as infection and major bleeding.
Heffern et al	USA	2021	Retrospective Chart Review	N = 36 who underwent reconstruction for penetrating facial trauma--> Subjects were categorized into four groups based on the subunit that underwent reconstruction: Involved subunits include 24 mandible,	range from 12-65 years, average 36.2	predominate mechanism of injury was self-inflicted GSW (n¼26, 72.2%). The other 10 subjects (27.8%) were victims of assault	Not specified, although one mention of patient injured by AK-47	timing of reconstruction only assessed for cranium reconstruction group: classified as primary (n=2, within 7days of presentation) and delayed (n=9, after 7days). Otherwise NA	NA	Predominate reconstruction method was open reduction internal fixation only for mandible (45.8%), bone grafting for malar complex (81.8%), implant for orbit (66.7%) and cranium (63.6%), and local tissue rearrangement for palate (84.6%)<br>N = 24 of 36 (66.7%) required mandible reconstruction. 6 of 24 (25.0%) subjects underwent primary external fixation of the mandible before definitive reconstruction. Definitive reconstruction consisted of:	NA	NA

				11 malar complex, 13 palate, 18 orbit, and 11 cranium						- ORIF alone in 11 of 24 (45.8%) subjects<br>- bone grafting with ORIF in 8 of 24 (33.3%) (14 bone grafts were used among the 8 subjects that underwent bone grafting for mandible reconstruction. The source of the bone grafts included iliac (n=6, 42.9%), fibula (n=2, 14.3%), mandible ramus (n=3, 21.4%), and allograft (n=3, 21.4%)		
Gurunluoglu and Gatherwright	USA	2018	Retrospective Case Control	patients receiving free flaps for early reconstruction = 24, patients receiving free flaps for delayed reconstruction = 2	41.3	self-inflicted, assault in United States	handgun (majority), shotgun, assault rifle	All initial free tissue procedures were completed within 1-2 weeks after the injury. Timing regarding revisions included the interval between free flap reconstructive surgeries which was kept relatively short (preferably few days - 1 week) to avoid scar tissue and contracture secondary to fast healing.	mean = 17.2 months (3-96 range)	Exploration, debridement, repair of soft tissue and/or facial fractures, bone stabilization using plates, maxillomandibular fixation were carried out during the initial operative stage, whenever possible, concomitantly with neurosurgical procedures. Also, local or regional flaps were performed for less severe injuries. Reconstruction of composite mandible defects was done with the osteocutaneous fibula flap; lip injuries were done with local flaps when appropriate. Secondary free flaps were typically needed for near-total/total full-thickness lower lip loss.<br><br>No single reconstructive method met the needs of all maxillofacial GSW patients and defects; each case depended on the location and requirements of the defect, available donor sites, as well as surgeon preference and experience.	The GSW patients indicated 60% satisfaction with appearance, along with 90% showing perceptible speech and 100% oral competence.	Complication rate was 45.2% and included: soft tissue infection = 5, orocutaneous fistula = 5, wound dehiscence = 3, partial flap loss = 3, nonunion = 3, osteomyelitis = 1, brain abscess = 1, and oral incompetence = 1. Performing two free flaps did increase the risk of complications which, we believe, could be attributed to complexity of injuries and reconstruction.
Shackford et al	USA	2014	Retrospective Cohort	patients with GSWBIF = 720	only median given = 29	assault in United States	handgun, rifle, shotgun/explosive, unknown GSW	Urgent measures were all done with 48 hours.	Not specified, patients were organized by mortality within 48 hours and otherwise.	Urgent measures within 48 hours of admission included endotracheal intubation (92% of patients), nasotracheal intubation (2.5%), cricothyroidotomy (5%), and tracheostomy (0.4%). Control of bleeding through balloon tamponade, angioembolization, and anterior/posterior nasal packing were also used. Urgent airway control's need was infrequent.<br><br>Total operations were significantly associated with complications of care, with an average of 5.4 operations per patient.	Aesthetics were not commented on, except that further investigation is needed.	Mortality rate was 26%, complication rate was 38%. Complications included blindness, infection, cranial nerve defects, pulmonary (pneumonia, atelectasis, pneumothorax, acute lung injury), nonunion/malunion, CVA, VTE, orocutaneous fistula, flap failure, seizures, cardiac (MI, arrhythmias), secondary hemorrhage, cerebrospinal fluid leak, and multiple organ failure.
Hollier et al	USA	2001	Retrospective Chart Review	N=84 with facial GSWs	Range 6-64 years, mean 27 years	Assault in US (54/84 suffered a single gunshot wound and 30/84 suffered multiple gunshot wounds), self-inflicted (N=5)	NA	NA	Mean hospital stay was 8.3 days, with a mean follow-up of 22 weeks. However, 40% of patients did not return for follow-up. The shortest follow-up was one week and the longest was 29 months	With respect to bony injuries, surgical repair was required from 32% to 76% of the time, depending on the injury. **other surgeries not specified except passively in figure captions with no clear data correlate, no further details on surgery type or timing**<br><br>Number injured/number requiring surgery<br>Zygoma injury n=29/10<br>Mandible injury n=25/19 (open reduction and internal fixation, placement of an external fixator, or the application of maxillomandibular fixation)<br>Orbit injury n=22/7<br>Skull injury n=18/7<br>Nasoethmoid n=10/5	subjective statement, however no supporting data reported:<br><br>"facial gunshot wounds are associated with a very high incidence of injuries requiring surgical intervention. It is our belief that these injuries should be addressed early, with procedures designed to	The complications resulting from the facial gunshot wounds (not all related to surgical intervention):<br><br>Early complications:<br>-visual disturbances =10<br>-wound dehiscence n=4<br>-generalized sepsis n=2<br>-stroke n=1<br>-speech difficult n=1<br>-CSF leak n=1<br>-facial nerve palsy

											repair both bone and soft tissue injuries simultaneously"	n=1<br>-seroma n=1<br>-acute renal failure n=1<br>-DIC n=1<br><br>Residual Problems:<br>-Cranial nerve palsy n=16<br>-blindness n=14<br>-hemiparesis n=10<br>-mental/psych n=4<br>-mandible n=4 (not sure what this means)<br>-epiphora n=2<br>-ptosis n=1
Maxwell et al	USA	2022	Case Series	42 patients with temporal bone ballistic injury	30.3 (5-58)	assault in USA	shotgun, otherwise not specified	Four patients were decompressed in the acute period (after medical stabilization and within 3 weeks of injury), and one attempt was made in a chronic setting (1 year after injury), which was unsuccessful (the surgeon was unable to localize the facial nerve at the stylomastoid foramen due to extensive scarring and nerve degeneration). No attempts to decompress the nerve were performed in an intermediate setting.	Range of 1 day to 9 years.	Transmastoid facial nerve decompression to remove compressive bony spicules (n = 5); eye protection surgery (n = 3); and peripheral facial nerve exploration (n = 1), noting transection at the pes. One required middle cranial fossa and transmastoid repair of cerebrospinal fistulae in setting of severe meningitis.<br><br>Transmastoid nerve decompression improved HB sore in 80% of patients.	N/A	Had difficulty finding specific complications noted
Saad et al	USA	2013	Case Series	patients who underwent complex maxillofacial reconstruction using virtual surgical planning = 10	53 + 9	not specified for one, other was self-inflicted	not specified	Timing was not specified for first surgery. In one case, patient had undergone multiple reconstructive procedures at other institutions within 5 years since his initial injury.	3 months	Reconstructive procedures included multiple nonvascularized bone grafts and two free forearm flaps, followed by virtual surgical planning for a free fibula osteocutaneous flap. Anterior projection of the midface was accomplished with the bony and soft-tissue reconstruction. The oronasal fistula was repaired with the skin island.<br><br>Virtual surgical planning proved effective in reconstruction of maxillary defects as well, and is suited to situations where both maxilla and mandible need reconstruction. The authors noted that although the use of simultaneous free flaps for massive oromandibular defects has been described, the optimal functional reconstruction of simultaneous maxillary and mandibular defects is not well known.	Initially, patients had issues with aesthetic outcomes. The procedures described with the virtual surgical planning showed improvement in ability to eat oral diet and stable bony framework. All flaps were viable at end follow-up and all patients had functional mandibular range of motion.	There were no intraoperative complications noted. Wound infection occurred in one of the GSW patients.
Pereira et al	USA	2012	Retrospective review	N = 28 who sustained GSWs to the face and required operative intervention (also compares injury pattern to MVA but excluded this data below)	28 +- 8.3	NA	single projectiles from low-velocity handguns predominated (no further specification)	Most patients presented within a few hours of the injury, only 1 appearing after 24 hours-- did not specify timing of surgery or # of surgeries/patient	The average hospitalizatio n was 8.3 (+- 7.1) days. Fifty percent of cases required intensive care unit (ICU) admission. The mean stay in the ICU was 5.2	After debridement, irrigation and the removal of foreign bodies (teeth, bone fragments and metallic objects) are performed; reduction and stabilization of skeletal injuries, and the provision of soft-tissue closure was addressed.<br><br>All 28 patients requiring operative intervention had facial fractures (85.8% patients with mandibular fractures). In the 28 operative cases, injuries predominantly involved the lower face, with open reduction and internal fixation (ORIF) of the mandible	NA	Tracheostomy was required in 35.7% of cases, but in only 2 was this emergent and awake

									(+-5.7) days. no further FU infomration	being the most frequent operative intervention (68%). Maxillary ORIFs were somewhat less common, accounting for 10.7%. External fixation required in 1 of 28 patients. MMF required in ~50% patients (had to interpret from bar graph so number not exact).<br><br>Free tissue transfer for reconstruction was required in 14.2% cases (3 fibular free flaps and 1 TRAM— 2 of these were fibular free flaps for mandibular reconstruction— these were not carried out acutely, but timing not specified).<br><br>timing of surgery or # of surgeries per patient not reported		
Motamedi	Iran	2011	Case Series	N=30 underwent primary debridement and arch bar placement followed by open reduction of fractures (with or without osteosynthesis) and primary wound closure. (Primary early intervention was done when there was no gross infection, no bone comminution or extensive soft tissue avulsion (precluding wound coverage), and when general health, concomitant injuries requiring more urgent attention or those requiring major grafts did not preclude this	24.4±7.8	NA	NA	"acute/early" treatment, although exact timing of primary surgery and revision sugeries are not described	NA	N= 30 acute management directed toward early treatment of both hard and soft tissue injuries in the first operation:<br>62.5% - Primary debridement + open fracture reduction (without wire, plate, or screw osteosynthesis) + wound closure<br>37.5% - Primary debridement + open reduction (wire, plate, or screw osteosynthesis) + wound closure<br><br>The mandible was injured in 96% and the maxilla in 54%<br>64% were managed in a single definitive early operation and 36% required two major operations. Revisions and secondary operations (in other areas) were performed in 36% of the patients.	restoration of occlusion and continuity of the jaw, fixation of luxated teeth, early return of function, prevention of segment displacement and tissue contracture, less scarring, and decreased need for major bone graft reconstruction. Flap healing was favorable in all patients.	22% required tracheostomy and 6/30 patients had minor complications such as scarring and wound discharge (transient postoperative discharge from the flap suture site, resolved within several weeks following irrigation and cleansing). No patients had major complications (i.e., necrosis or osteomyelitis).
Jeyaraj and Chakranarya n	India	2018	Retrospective Cohort	patients who underwent an early, aggressive, surgical intervention = 20, patients who underwent resuscitation and primary soft tissue closure followed by conservative, closed reduction techniques, delayed repair, and late reconstruction of bone soft tissue defects = 20	not specified	Combat in Middle East	not specified	For early aggressive, one/single-stage definitive management with immediate reconstruction, the first major surgery was performed within the first 48 h, and the reconstructive surgery to manage hard tissue, soft tissue or composite defects within the first 7 days.<br><br>For the combined phase-approach, definitive reconstruction is done as early as possible, with esthetic/functional refinements occurring weeks to months with free flap debunking and contouring.	not specified	Patients were divided into two groups: one who had undergone an early, aggressive, surgical intervention, by ORIF of fractured bones of the maxillofacial skeleton, employing mini and microplate fixation techniques with/without immediate bone grafting and the other who were managed using conservative, closed reduction techniques, followed by delayed repair and late reconstruction of bone defects<br><br>Early, definitive, and aggressive maxillofacial surgical techniques proved superior to the conservative approach by bringing about primary bone healing and minimizing residual deformities and subsequent scar contractures, thus yielding improved functional as well as superior esthetic outcomes.	Early technique showed better healing and fewer and less complex surgical revisions than the patients who have undergone delayed surgical reconstructions and overall improved ultimate esthetic and functional outcomes.	Wound site infection, delayed healing, non-/mal-union, residual deformities, and scar contractures.
Chaiyasate et al	USA	2022	Case Series	(Total of 13 patients underwent 23 free flaps). Patients with	average age = 26, median =	11 self-inflicted, 2 assaults	not mentioned	24 to 48 hours - initial<br>6 weeks to 3 months	3.3 (1.3–3.9) years	Avoiding early use of reconstruction plates, establishing occlusion early, and aligning bony segments using external fixation.	Patients can return to normal life and have	

				fibular free flap = 14, patients with radial forearm free flap = 6, patients with scapula free flap = 2, patients with medial femoral condyle flap = 1.	21, IQR = [17,37]			- Reconstruction<br>>9 months (>6 months after last surgery) - Revision surgery		Aggressive early reduction of the fracture and reestablishment of the facial buttress followed by delayed free flap reconstruction is the preferred method of facial reconstruction<br><br>Designed an algorithm for treating different regions and timing of 0 days - Gun shot wound<br>24 to 48 hours - ATLS protocol, tracheostomy / G tube, 3D CT and CT angiogram --> Early conservative debridement of soft tissues, Meticulous soft tissue repair, Aggressive debridement of bone fragments, Midface ORIF with miniplates, Mandible open reduction with external fixators<br>6 weeks to 3 months - Recovery period allowing soft tissue to heal / family counseling / Social worker / addressing associated injuries / preparation for multi- stage craniofacial reconstruction with CAD /CAM--> Delayed Definitive Craniofacial Reconstruction (proposes options for different regions listed below)<br>>9 months (>6 months after last surgery) - Revision surgery	"acceptable" aesthetic outcomes	
Tahmasebi et al	Iran	2024	Prospective Cohort	Patients with fibula flap treatment = 14 (10 bone only, 4 had osseo-cutaneous components)	34.93 + 5.17 years (age range: 27-42 years)	Not explicitly stated	Not stated	Reported value does not make sense (Delay between trauma and surgery 14.102 + 86.24, range: 90-180)	6 months	Systolic blood pressure (p<0.001), diastolic blood pressure (p<0.001), and oxygen saturation percentage (p=0.001) were significantly higher in successful flap cases.<br><br>Smoking reduced flap success<br><br>Non predictors of success: site of arterial anastomosis, type of artery, and anastomosis vein at the recipient site, location of defects, gender, and time between trauma and surgery<br><br>Flap success 78.6% (n = 11)		infection - 5 patients (35.7%)<br>hematoma - 1 patient (7.1%)<br>thrombosis - 5 patients (35.7%)<br>bleeding - 2 patients (14.3%)
Peled, et al	Israel	2011	Retrospective Case Review	22 Patients	25.9 years	2006 Israel Uprising	GSW, Blast Shrapnel	Immediate upon transport to military hospital	4-54 days	Irrigation, Debridement, tissue preservation, plate fixation<br><br>Average 1.5 procedures per\ patient	Not Discussed	Infection - 1<br>Enophthalmos - 1
